# Design characteristics of studies evaluating the effect of non‐surgical periodontal treatment on systemic health outcomes

**DOI:** 10.1002/JPER.24-0847

**Published:** 2025-11-19

**Authors:** Timothy Treat, Dylan Jones, Natalie Lorenzano, Scott Umberfield, Andrew Bartels, Titus Schleyer, Heather Taylor

**Affiliations:** ^1^ Indiana University School of Dentistry Indianapolis Indiana USA; ^2^ University of Iowa College of Dentistry and Dental Clinics Iowa City Iowa USA; ^3^ Richard L. Roudebush VA Medical Center Indianapolis Indiana USA; ^4^ Regenstrief Institute Indianapolis Indiana USA; ^5^ Indiana University School of Public Health Indianapolis Indiana USA

**Keywords:** biomarkers, inflammation, periodontal disease, periodontal medicine, treatment outcome

## Abstract

**Background:**

Evidence for whether and how non‐surgical periodontal treatment (NSPT) improves systemic outcomes remains equivocal. Inconclusive findings may in part be due to design variations of randomized controlled trials (RCTs) in this field. The objective of this study was to describe the study design characteristics of RCTs that have evaluated the effect of non‐surgical periodontal treatment on systemic health outcomes.

**Methods:**

We searched Medline via Ovid and EMBASE for RCTs published through January 1, 2024, for terms indicating NSPT (i.e., scaling and root planing) and selected chronic diseases. We developed a standardized coding sheet for systematically extracting data from studies, including the definition of periodontal disease (PD) among study participants, the length of study duration, and whether the effect of NSPT was found to have a statistically significant beneficial, detrimental, or null effect on systemic outcomes.

**Results:**

Eighty‐two RCTs, which reported the effect of NSPT on systemic outcomes in 816 individual analyses, met our inclusion criteria. Fifty‐six studies (68.3%) had at least 1 of 4 variations in study design that may contribute to biased results. Studies that restricted their inclusion criteria to participants with severe PD were more likely to measure a beneficial effect than a non‐beneficial effect on specific systemic outcomes following NSPT (62.7% vs. 49.2%, *p* < 0.001).

**Conclusion:**

Variation in study designs of RCTs may be contributing to mixed and inconclusive findings investigating the effect of NSPT on systemic disease outcomes.

**Plain language summary:**

This study reviewed clinical trials to understand why the evidence on whether non‐surgical periodontal treatment (NSPT) can improve overall health is inconclusive. Eighty‐two clinical trials were analyzed to identify patterns in how these studies were designed. While most trials found that NSPT had some benefits, more than two‐thirds of the studies had design features that could skew results. Trials involving participants with severe gum disease were more likely to show benefits than those including people with milder forms. How clinical trials are set up—such as who is included and how long the study lasts—may heavily affect overall findings. The study highlights the need for more standardized approaches to research in this area to better understand whether and how dental treatments can improve overall health. These findings are important for designing future studies and ensuring reliable evidence for medical and dental professionals.

## 1 INTRODUCTION

Periodontal disease (PD) is a significant public and population health problem, with over 42% of US adults (> 30 years old) having some form of PD and 8% having severe PD.^1^ PD destroys tissue and bone supporting teeth, potentially leading to infection, loss of function, and, ultimately, tooth loss.^2^ Given that PD negatively affects quality of life and is associated with other common systemic diseases and conditions,^3^, ^4^, ^5^, ^6^, ^7^, ^8^ it is important to treat and manage the disease effectively. Beyond improving oral health outcomes, the treatment of PD may also improve systemic health outcomes by reducing local inflammation caused by PD, and subsequently reducing the host's overall inflammatory burden.^7^, ^9^ However, evidence for whether and how periodontal interventions improve systemic outcomes remains equivocal.^8^

Whether periodontal treatment positively affects systemic outcomes remains inconclusive in part due to the underlying research methodology and rigor of the studies that examine this relationship.^8^ Systematic reviews have attributed the mixed findings to significant differences in the design and approach of clinical studies, including variability in PD definitions, length of study durations, and overall low sample sizes.^8^, ^10^, ^11^, ^12^ Additionally, how periodontal treatment is delivered within these clinical studies may be inconsistent across patient populations, adding to the elusiveness of conclusive findings. These sources of variation can limit researchers’ ability to combine analyses and generate policy implications. For instance, measuring a diabetes‐related systemic outcome, such as glycated hemoglobin (HbA1c), at 3 months versus 6 months post‐periodontal treatment may impact the observed effect. Similarly, the effect of periodontal treatment on HbA1c may be modulated by the level or severity of PD among the study participants (e.g., mild vs. severe PD). If wide variations exist across studies examining the relationship between PD treatment and systemic outcomes, then our ability to draw conclusions from aggregated data in systematic reviews and meta‐analyses is limited and potentially biased. In consequence, our ability to inform best clinical practices, policy, and population health interventions is also limited. To address these differences across studies and make future improvements in study designs, we must first describe the evidence that links periodontal treatment and systemic outcomes and identify the variations in the designs of these studies that may explain why findings remain mixed across randomized controlled trial (RCTs) and inconclusive across systematic reviews and meta‐analyses in this field.

Thus, the purpose of this study was to characterize design features of RCTs that have evaluated the effect of non‐surgical periodontal treatment (NSPT) on systemic outcomes. By assessing commonalities and differences across these studies, we aim to provide insights for future research on the oral‐systemic health link, including the design and methodological approach of RCTs. These findings may be of interest to clinical researchers, as well as medical and dental providers who, respectively, manage patients with multiple co‐morbidities.

## 2 MATERIALS AND METHODS

### 2.1 Protocol

Although this study is descriptive, our protocol was developed a priori, and the search strategy was informed by the Preferred Reporting Items for Systematic Reviews and Meta‐Analyses (PRISMA) guidelines.^13^

### 2.2 Search strategy

To identify RCTs that have focused on the relationship between NSPT and systemic disease and condition outcomes, we searched 2 bibliographic databases (Medline via Ovid and EMBASE) for RCTs published through January 1, 2024. Terms such as *periodontics, dental prophylaxis, pregnancy, cardiovascular diseases (CVDs), diabetes mellitus, rheumatoid arthritis (RA), obesity, and chronic kidney disease (CKD)* were included in our keyword search and modified based on the database searched. Tables S1 and S2 in the online *Journal of Periodontology* include the full list of search terms for each database. We did not search the gray literature or trial registries, as the focus of this study was on evidence provided through peer‐reviewed RCTs.

### 2.3 Inclusion/exclusion criteria

To be included, a study had to be (1) a peer‐reviewed RCT, (2) written in English, and (3) focused on the effect of NSPT on outcomes of a systemic disease or condition in adults. Although there are several treatments used to manage PD, we focused on NSPT interventions (i.e., scaling and root planing) because this treatment is the most common approach employed by dental professionals.^8^, ^14^, ^15^ We excluded studies whose primary intervention arm in the treatment group included other less common treatment modalities for PD, such as lasers and antibiotics. Finally, we excluded any RCT that evaluated the effect of NSPT on *only* periodontal outcomes of patients with systemic diseases.

### 2.4 Study selection

**FIGURE 1:**
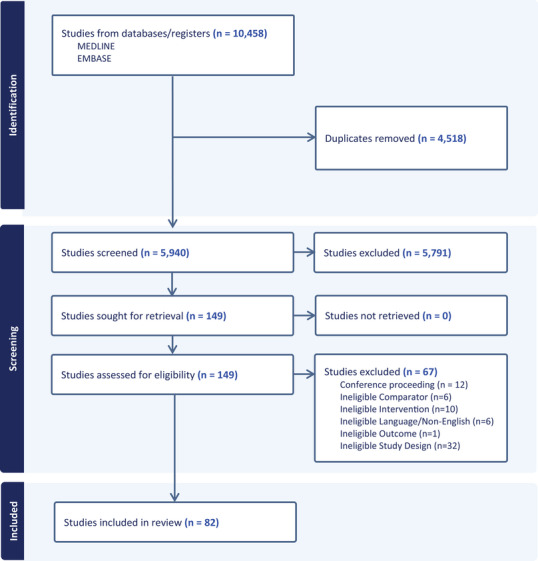
Study identification and selection process. Preferred Reporting Items for Systematic Reviews and Meta‐Analyses (PRISMA) flow diagram illustrating article screening process. Of 10,458 articles retrieved from the search strategy, a total of 82 were selected for inclusion in this study.

To determine whether to include a study in the review, 4 reviewers (T.T., N.L., D.J., H.T.) independently examined the title and abstract of all articles obtained from the combined database searches. Subsequently, the full text of articles was reviewed in detail against the inclusion/exclusion criteria. Each article was screened by at least 2 reviewers. Any disagreements among reviewers were resolved in team discussion. Figure 1 documents our article selection process; Table S3 in the online *Journal of Periodontology* lists excluded studies and reasons for their exclusion; and Table S4 in the online *Journal of Periodontology* contains the full list of included studies.

### 2.5 Data extraction

A standardized coding sheet was developed a priori (see Figure S1 in the online *Journal of Periodontology*) to systematically extract relevant data from each study in 2 phases. In the first phase, 6 reviewers (T.T., S.U., A.B., D.J., N.L., H.T.) independently completed the coding sheet for 10 (12.2%) of the included studies. The reviewers then met to reach consensus and make any necessary adjustments to the data extraction process and coding sheet. In the second phase, reviewers split into teams of 2 to extract data from the remaining studies. Thus, for each study, 2 reviewers independently reviewed and extracted data. Any conflicting findings were resolved through discussion between the 2 assigned reviewers or by consultation with the entire team of reviewers.

From each included RCT, we extracted study design characteristics including the study population; details on the definition, severity, and extent of PD; information on study group interventions; outcomes evaluated; and the study duration. Based on the full text review and coding sheet results, we classified 3 study design characteristics as potential sources of variation that may have led to biased results: (1) missing severity or stage of PD; (2) any instance of the control group receiving treatment that could contribute to reduced levels of inflammation in the mouth, such as dental extractions or a prophylaxis; and (3) not accounting for the smoking status of study participants.

Similar to previous work,^8^ data on empirical results of all outcomes reported within each study were coded as either: (1) a “beneficial effect” if a study reported a statistically significant positive association for positive outcomes (e.g., higher birth weight) and negative association for negative outcomes (e.g., lower rate of preterm birth), (2) an “adverse effect” if a study reported a statistically negative association for positive outcomes (e.g., lower birthweight) or positive associations for negative outcomes (e.g., higher HbA1c), or (3) “null” if no statistically significant differences were reported.

### 2.6 Analyses

We used a web‐based software* for managing our article selection processes, including combining search results, removing duplicates, and selecting articles for full‐text review. Data extracted from final consensus coding sheets were entered, cleaned, and prepared for analysis.

As part of the analysis, means and frequencies of various study characteristics by study disease focus were tabulated. Next, potential variations in the design of included studies and the proportion of studies with each type of variation were described. To analyze the bivariate relationship between the severity level of PD and beneficial study conclusions, we conducted Chi‐square tests with a *p*‐value of < 0.05 indicating statistical significance. Finally, to inspect how study duration may affect results, we plotted the effect of NSPT on systemic outcomes that appeared in 3% or greater analyses by the reported study durations. Given that adverse birth outcomes were consistently measured at the point of birth or spontaneous abortion, and not measured at specific predetermined time periods following NSPT, we separately plotted adverse birth outcome effects that appeared in 3% or greater analyses by the trimester when the NSPT intervention was administered to the mother.

## 3 RESULTS

The search strategy retrieved 10,458 studies from Medline via Ovid and Embase (see Figure 1). After duplicates were removed, 4518 titles and abstracts were screened, resulting in 149 studies considered for full‐text review. Of these, 82 studies met our inclusion criteria, including 32 focused on diabetes,^16^, ^17^, ^18^, ^19^, ^20^, ^21^, ^22^, ^23^, ^24^, ^25^, ^26^, ^27^, ^28^, ^29^, ^30^, ^31^, ^32^, ^33^, ^34^, ^35^, ^36^, ^37^, ^38^, ^39^, ^40^, ^41^, ^42^, ^43^, ^44^, ^45^, ^46^, ^47^ 19 on adverse birth outcomes,^48^, ^49^, ^50^, ^51^, ^52^, ^53^, ^54^, ^55^, ^56^, ^57^, ^58^, ^59^, ^60^, ^61^, ^62^, ^63^, ^64^, ^65^, ^66^ 20 on CVD,^67^, ^68^, ^69^, ^70^, ^71^, ^72^, ^73^, ^74^, ^75^, ^76^, ^77^, ^78^, ^79^, ^80^, ^81^, ^82^, ^83^, ^84^, ^85^, ^86^ 4 on CKD,^87^, ^88^, ^89^, ^90^ 3 on RA,^91^, ^92^, ^93^ 3 on obesity,^94^, ^95^, ^96^ and 1 on lupus.^97^ A total of 816 individual systemic outcomes were examined within the included studies (see Table 1). The sample size ranged from 14 to 1806, with an average of 182 participants. Studies focusing on adverse birth outcomes had the highest average number of participants (*n* = 427 participants) followed by studies focused on CVD outcomes (*n* = 124 participants).

**TABLE 1:** Overall characteristics of randomized controlled trial studies evaluating the effect of non‐surgical periodontal treatment (NSPT) on systemic outcomes (*n* = 82).

Parameter	All Studies *N* = 82	ABO^a^ *N* = 19	CVD^b^ *N* = 20	Diabetes *N* = 32	CKD^c^ *N* = 4	RA^d^ *N* = 3	Obesity *N* = 3	Lupus *N* = 1
No. of systemic outcomes analyzed	816 (100%)	130 (15.9%)	298 (36.5%)	237 (29.0%)	65 (8.0%)	24 (2.9%)	59 (7.2%)	3 (0.4%)
Average sample size (SD)	181.7 (277.1)	427.4 (477.2)	124.3 (96.1)	109.3 (116.1)	66.8 (41.6)	49.3 (28.7)	95.7 (54.0)	90.0 (‐)
Sample size range	14–1806	20–1806	22–317	23–514	14–100	28–82	50–158	–
Participant sex specified in study								
Yes	80 (97.6%)	19 (100%)	19 (95.0%)	31 (96.9%)	4 (100%)	3 (100%)	3 (100%)	1 (100%)
No	2 (2.4%)	–	1 (5.0%)	1 (3.1%)	–	–	–	–
Participant race specified in study								
Yes	34 (41.5%)	14 (73.7%)	8 (40.0%)	8 (25.0%)	1 (25.0%)	1 (33.3%)	2 (66.7%)	‐
No	48 (58.5%)	5 (26.3%)	12 (60.0%)	24 (75.0%)	3 (75.0%)	2 (66.7)	1 (33.3%)	1 (100%)
Study reported funding								
Yes	66 (80.5%)	13 (68.4%)	16 (80.0%)	27 (84.4%)	4 (100%)	2 (66.7%)	3 (100.0%)	1 (100%)
No	16 (19.5%)	6 (31.6%)	4 (20.0%)	5 (15.6%)	–	1 (33.3%)	–	–
Extent of periodontal disease among participants								
Both	2 (2.4%)	–	–	2 (6.3%)	–	–	–	–
Generalized	25 (30.5%)	3 (15.8%)	5 (25.0%)	9 (28.1%)	4 (100%)	2 (66.7%)	2 (66.7%)	–
Localized	3 (3.7%)	2 (10.5%)	–	1 (3.1%)	–	–	–	–
Not specified	52 (63.4%)	14 (73.7%)	15 (75.0%)	20 (62.5%)	–	1 (33.3%)	1 (33.3%)	1 (100%)
PD state of study participants								
Chronic	41 (50.0%)	3 (15.8%)	9 (45.0%)	21 (65.6%)	4 (100%)	2 (66.7%)	2 (66.7%)	–
Acute	–	–	–	–	–	–	–	–
Not specified	41 (50.0%)	16 (84.2%)	11 (55.0%)	11 (34.4%)	–	1 (33.3%)	1 (33.3%)	1 (100%)
Severity/stage of PD among study participants								
Not defined	29 (35.4%)	12 (63.1%)	6 (30.0%)	10 (31.3%)	–	–	1 (33.3%)	–
Initial PD/ stage I	–	–	–	–	–	–	–	–
Moderate PD/ stage II	11 (13.4%)	4 (21.1%)	1 (5.0%)	5 (15.6%)	–	1 (33.3%)	–	–
Severe PD/ stage III & IV	34 (41.5%)	1 (5.3%)	12 (60.0%)	14 (43.8%)	3 (75.0%)	1 (33.3%)	2 (66.7%)	1 (100%)
Included all stages	8 (9.8%)	2 (10.5%)	1 (5.0%)	3 (9.4%)	1 (25.0%)	1 (33.3%)	–	–
Study duration								
1.5 months	1 (1.2%)	–	1 (5.0%)	–	–	–	–	–
2 months	4 (4.9%)	–	2 (10.0%)	1 (3.1%)	–	1 (33.3%)	–	–
3 months	21 (25.6%)	–	6 (28.6%)	11 (34.4%)	1 (25.0%)	1 (33.3%)	1 (33.3%)	1 (100%)
4 months	3 (3.7%)	–	–	3 (9.4%)	–	–	–	–
6 months	31 (37.8%)	–	9 (45.0%)	16 (50.0%)	3 (75.0%)	1 (33.3%)	2 (66.7%)	–
12 months	3 (3.7%)	–	2 (10.0%)	1 (3.1%)	–	–	–	–
Term of pregnancy	19 (23.2%)		–	–	–	–	–	–
1st trimester		11 (57.9%)						
2nd trimester		7 (36.9%)						
3rd trimester		1 (5.3%)						
Overall study conclusion								
NSPT is beneficial	53 (64.6%)	8 (42.1%)	14 (70.0%)	24 (75.0%)	2 (50.0%)	3 (100%)	1 (33.3%)	1 (100%)
No difference in effect	29 (35.4%)	11 (57.9%)	6 (30.0%)	8 (25.0%)	2 (50.0%)	–	2 (66.7%)	–
NSPT is detrimental	–	–	–	–	–	–	–	–

Abbreviation: PD, periodontal disease.

^a^ Adverse birth outcomes.

^b^ Cardiovascular disease.

^c^ Chronic kidney disease.

^d^ Rheumatoid arthritis.

*Source*: Primary data collection from Medline and Embase databases up to December 2023.

Overall, 53 studies (64.6%) concluded that NSPT was beneficial and improved systemic outcomes, while 29 studies (35.4%) reported no statistically significant effects. No study reported that NSPT was detrimental to systemic outcomes. More specifically, all studies with a focus on participants with RA (*n* = 3; 100%) and lupus (*n* = 1; 100%) reported beneficial impacts on systemic outcomes following NSPT. In contrast, the majority of RCTs investigating the effect of NSPT on adverse birth outcomes (*n* = 11; 57.9%), and obesity (*n* = 2; 66.7%) reported no significant effect on systemic outcomes.

Overall, 56 studies (68.3%) had at least 1 variation in study design that may have contributed to biased results (see Table 2). First, in 29 studies (35.4%), the severity of PD among study participants was not documented or reported. Second, in 9 studies (11.0%), the control group received dental extractions. Third, in 24 studies (29.3%), the control group received supragingival scaling or prophylaxis as part of their assigned intervention. Fourth, 8 studies (9.8%) did not report, account for, or balance the smoking status of study participants across groups.

**TABLE 2:** Study design characteristics that may contribute to biased results among included studies (*n* = 82).

Study design characteristics	All Studies *N* = 82	ABO^a^ *N* = 19	CVD^b^ *N* = 20	Diabetes *N* = 32	CKD^c^ *N* = 4	RA^d^ *N* = 3	Obesity *N* = 3	Lupus *N* = 1
Severity or stage of periodontal disease among participants unspecified	29 (35.4%)	12 (63.2%)	6 (30.0%)	10 (31.3%)	–	–	1 (33.3%)	–
Control group received tooth extractions	9 (11.0%)	3 (15.8%)	2 (10.0%)	3 (9.4%)	–	–	1 (33.3%)	–
Control group received supra‐gingival scaling or prophylaxis	24 (29.3%)	6 (31.6%)	8 (40.0%)	9 (28.1%)	–	–	1 (33.3%)	–
Did not account for smoking status of study participants	8 (9.8%)	4 (21.1%)	–	3 (9.4%)	–	1 (33.3%)	–	–
Total no. of unique studies	56 (68.3%)	17 (89.5%)	14 (70.0%)	22 (68.8%)	0 (0.0%)	1 (33.3%)	2 (66.7%)	0 (0.0%)

*Note*: Some studies have more than 1 potential critical variation in design.

^a^ Adverse birth outcomes.

^b^ Cardiovascular disease.

^c^ Chronic kidney disease.

^d^ Rheumatoid arthritis.

*Source*: Primary data collection from Medline and Embase databases up to December 2023.

Table 3 presents the bivariate relationships between the severity level of PD among study participants and beneficial study outcomes. Studies that restricted their inclusion criteria to participants with severe PD were more likely to measure a beneficial effect on systemic outcomes following NSPT than a non‐beneficial effect (62.7% vs. 49.2%, *p* < 0.001). These observed bivariate relationships were not detected when comparing studies that included all stages of PD or were restricted to moderate PD. Overall, out of the 816 individual systemic outcomes reported across all timepoints in all included studies, NSPT had no significant beneficial effect on the majority of outcomes (*n* = 591; 72.4%).

**TABLE 3:** Bivariate relationships between beneficial study and outcome conclusions and the severity/stage of periodontal disease (PD) among study participants in randomized controlled trials evaluating the effect of non‐surgical periodontal treatment on systemic outcomes.

	Beneficial effect reported in overall study conclusion (*n* = 82)^a^				Beneficial effect found among individual outcomes (*n* = 816)		
Parameter	Yes	No	*p*‐value		Yes	No	*p*‐value
Severity/stage of PD not defined (*n* = 29 studies)	16 (30.2%)	13 (44.8%)	0.185	Severity/stage of PD not defined (*n* = 166 analyses)	40 (17.8%)	126 (21.3%)	0.261
Moderate PD (stage II) (*n* = 11 studies)	7 (13.2%)	4 (13.8%)	0.941	Moderate PD (stage II) (*n* = 107 analyses)	19 (8.4%)	88 (14.9%)	0.015
Severe PD (stage III & IV) (*n* = 34 studies)	23 (43.4%)	11 (37.9%)	0.631	Severe PD (stage III & IV) (*n* = 432 analyses)	141 (62.7%)	291 (49.2%)	0.001
All stages of PD (stage I–IV) (*n* = 8 studies)	7 (13.2%)	1 (3.5%)	0.154	All stages of PD (stage I–IV) (*n* = 111 analyses)	25 (11.1%)	86 (14.6%)	0.200
Total	53	29			225	591	

*Note*: No studies restricted participants to only mild/stage 1 PD. Results generated from chi‐squared tests with statistical significance set at *p*‐value <0.05.

^a^ Derived from general conclusion statement in study abstract.

**FIGURE 2:**
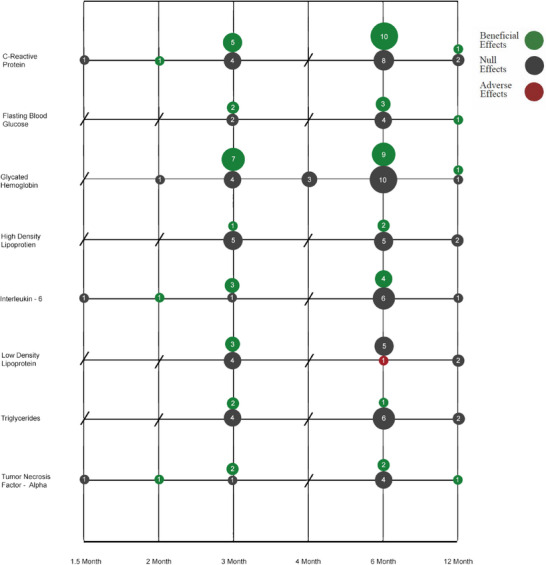
Effects of non‐surgical periodontal treatment on the most common systemic disease outcomes studied by study duration. The size of the bubble correlates to the number of analyses conducted at a certain time following non‐surgical periodontal treatment. Diseases studied include cardiovascular disease, chronic kidney disease, diabetes, obesity, rheumatoid arthritis, and lupus.

The most commonly measured systemic outcomes (see Figure 2) among studies that analyzed the effects of NSPT on CVD, CKD, diabetes, lupus, obesity, and RA were HbA1c (*n* = 36 analyses), C‐reactive protein (*n* = 32 analyses), interleukin‐6 (*n* = 17 analyses), high density lipoprotein (*n* = 15 analyses), low density lipoprotein (*n* = 14 analyses), triglycerides (*n* = 13 analyses), fasting blood glucose (*n* = 12 analyses), and tumor necrosis factor alpha (*n* = 12 analyses). Following NSPT, these outcomes were measured at the following 5 study duration time periods: 1.5 months (*n* = 3 analyses), 2 months (*n* = 4 analyses), 3 months (*n* = 48 analyses), 4 months (*n* = 3 analyses), 6 months (*n* = 80 analyses), and 12 months (*n* = 13 analyses). Of the total 153 analyses conducted on these systemic outcomes, 90 (58.9%) reported no difference in the outcome following NSPT, 62 (40.5%) reported a beneficial effect of NSPT on the outcome, and 1 (0.6%) reported an adverse effect of NSPT on the outcome. Of the 61 analyses that reported a beneficial effect, 23 (37.7%) were observed at 3 months post‐NSPT and 30 (49.2%) at 6 months post‐NSPT.

**FIGURE 3:**
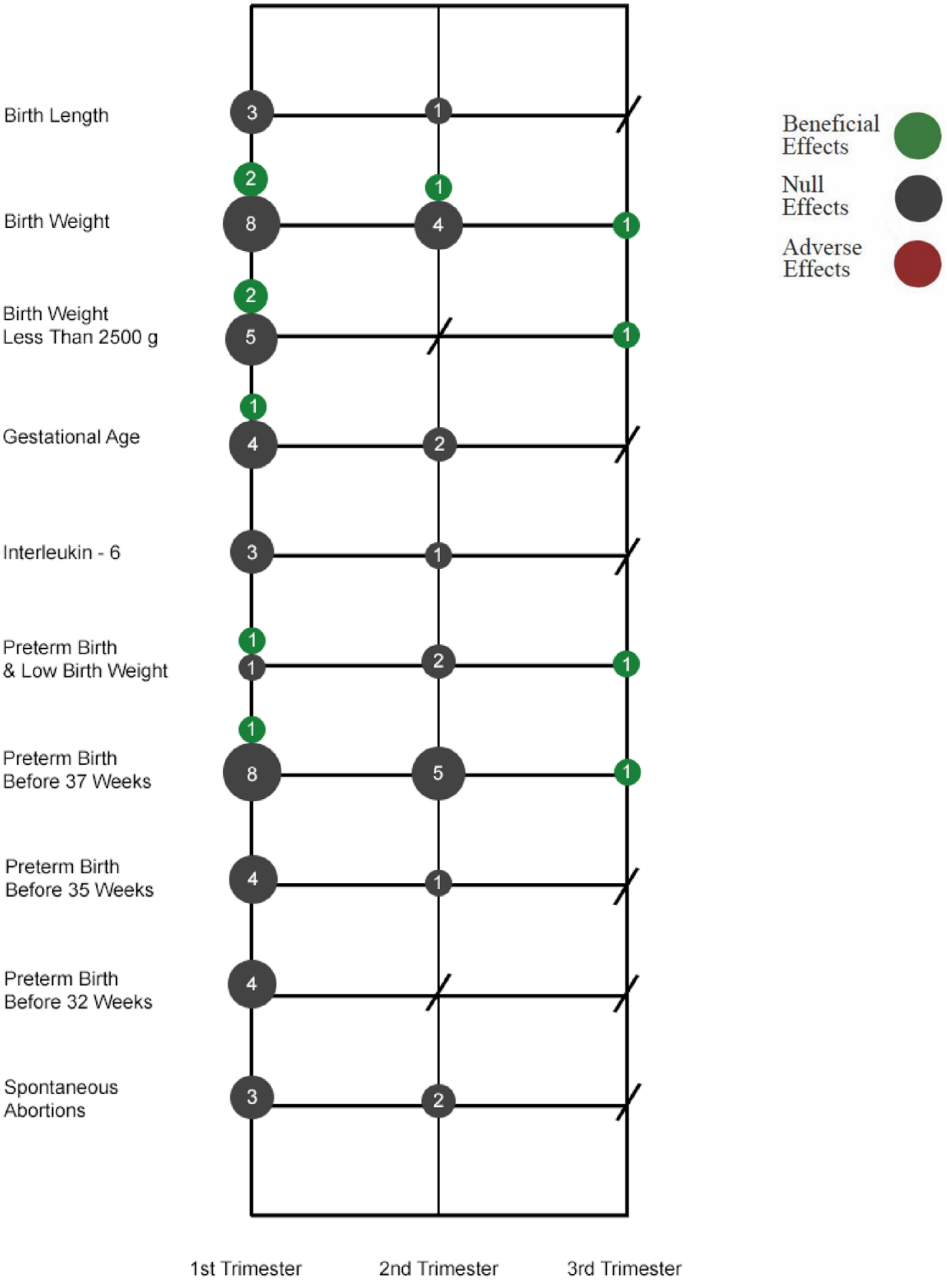
Effects of non‐surgical periodontal treatment on the most common adverse birth outcomes studied by trimester when treatment was administered. The size of the bubble correlates with the number of analyses conducted following non‐surgical treatment administered during a certain trimester.

Figure 3 presents the most commonly measured adverse birth outcomes by the trimester in which NSPT was administered in each study. These outcomes included total birth weight (*n* = 16 analyses), preterm birth before 37 weeks (*n* = 15 analyses), birthweight less than 2500 g (*n* = 8 analyses), gestational age (*n* = 7 analyses), preterm birth and low birth weight (*n* = 5 analyses), preterm birth before 35 weeks (*n* = 5 analyses), spontaneous abortion or stillbirth (*n* = 5 analyses), birth length (*n* = 4 analyses), interleukin‐6 (*n* = 4 analyses), and preterm birth before 32 weeks (*n* = 4 analyses). These outcomes were measured following NSPT delivered in the first trimester (*n* = 48 analyses), second trimester (*n* = 19 analyses), and third trimester (*n* = 4 analyses). Of the 73 analyses associated with these adverse birth outcomes, 12 (16.4%) reported a beneficial effect of NSPT on the outcome, and 61 (83.6%) reported no difference in the outcome following NSPT.

## 4 DISCUSSION

This study aimed to summarize evidence from RCTs that examine the relationship between NSPT and systemic disease outcomes while identifying variations in study design characteristics that may explain inconclusive findings in the field. The majority of RCTs included in this analysis reported that NSPT had an overall beneficial effect on systemic outcomes. However, caution is warranted given the considerable variation among results at the individual systemic outcome level and across various study design characteristics. When there is substantial variation in the approach of these studies, generating accurate and precise findings through aggregated data (i.e., systematic reviews/meta‐analyses), either within or across disease foci, becomes difficult if not impossible. Our findings underscore the equivocal and inconclusive nature of the field's current understanding of the relationship between NSPT and systemic outcomes. Our study highlighted that among study subjects with CVD, diabetes, CKD, RA, obesity, or lupus, there is no single outcome that consistently shows a beneficial response to NSPT (Figure 2). Null findings were more consistently observed among the most common birth outcomes measured following NSPT (Figure 3). Despite the continued ambiguity in this field, this study's results can be used to inform future research and improve the rigor of the scientific investigations of the oral‐systemic health link. To advance future research, the field would benefit from collaboration between experts in dentistry and medicine to establish a prioritized list of systemic outcomes and identify realistic follow‐up periods for detecting meaningful improvements in these chronic disease parameters.

The inclusion criteria for study participants appear to play a role in whether a significant beneficial effect is observed in systemic outcomes post‐NSPT. RCTs that enrolled participants with severe PD were more likely to find positive effects on systemic outcome measurements compared to studies that enrolled participants with moderate or all stages of periodontitis. This finding aligns with recent epidemiological work, which suggests that the relationship between uncontrolled diabetes and PD is primarily observed among those with *severe* periodontitis.^1^ Thus, the severity of PD may influence whether NSPT has a clinically measurable effect on systemic outcomes and may also explain why our understanding of the relationship between NSPT and systemic outcomes remains mixed across systematic reviews and meta‐analyses. Notably, over a third of RCTs did not adequately define or specify the severity or stage of PD among their study participants. Yet findings from these studies are often included in meta‐analyses and systematic reviews aimed at informing best practices and clinical guidelines.^8^ Previous research suggests that the definition of PD may contribute to different observed statistical associations between periodontitis and systemic disease/birth outcomes.^98^, ^99^ Thus, combining data across studies with varying or ill‐defined stages of disease severity may lead to biased results in aggregated data. Furthermore, the considerable variation in RCT sample sizes may also contribute to mixed findings in this field, as underpowered studies often lack the statistical strength to detect clinically meaningful effects in biomarkers.^100^

The 2017 World Workshop on the Classification of Periodontal and Peri‐Implant Diseases and Conditions, cosponsored by the American Academy of Periodontology (AAP) and the European Federation of Periodontology (EFP), updated the classification of PD to a multidimensional staging and grading system.^102^ While many of the included RCTs were conducted before this classification was published, future clinical trials should carefully document the severity, complexity, and extent of PD among study participants who receive NSPT. In doing so, results across studies can be more appropriately aggregated and analyzed by these PD characteristics. By adhering to these guidelines, data can be aggregated for meta‐analyses in ways that minimize heterogeneity across studies and enhance confidence in results. Moreover, since the beneficial systemic effects of NSPT may be attenuated by the severity level of PD among study participants,^98^, ^99^ clinical trials should explore systemic outcomes among populations with 1 specific severity level of PD.

Other study design approaches that may confound the relationship between NSPT and systemic outcomes include a lack of accounting for participant smoking status and providing control group participants with supragingival scaling, prophylaxis, or dental extractions as part of their assigned intervention. Given the substantiated relationship between smoking and PD and chronic conditions,^103^, ^104^, ^105^, ^106^ failure to report, account for, or balance the smoking status across study participant groups could lead to biased and inaccurate findings. Additionally, providing dental treatments like extractions or supragingival scaling to the control group may reduce oral inflammation among these participants and thereby lower systemic inflammatory markers. This could potentially confound the relationship between NSPT and measured systemic outcomes, particularly if the mechanism between PD and other chronic conditions is triggered by overall inflammation in the body. Some RCTs have addressed this by offering periodontal care to the control group after the study period was completed, ensuring that any systemic effects measured were not inadvertently attenuated while still delivering ethical care. Addressing these methodological challenges in study design is crucial for accurately assessing the impact of NSPT on systemic health outcomes. Future research should strive to account for smoking status among participants and carefully consider the timing and nature of interventions provided to control groups to minimize potential confounding effects.

Findings may also be affected by the length of time between NSPT and measured systemic outcomes. This current study presented evidence that considerable variation in study duration exists across RCTs, revealing gaps in our understanding of how systemic outcomes may be affected over time post‐NSPT. In some cases, systemic outcomes were measured a year after NSPT was performed, a timeframe that may exceed our ability to detect a clinically meaningful change. Given that clinical practice guidelines for management of PD include initial NSPT, re‐evaluation, and frequent recall visits for periodontal maintenance procedures,^107^ future research should align study durations with these common clinical practice intervals. Furthermore, because there is weak evidence regarding the timing of recall intervals,^108^, ^109^, ^110^, ^111^ identification of when beneficial systemic effects of NSPT occur and for how long they persist may advance the evidence base for setting personalized clinical recall intervals, particularly for patients with systemic health conditions.

Despite the strengths of this descriptive study, there are 4 important limitations to this work. First, our approach did not include an analysis of the risk for bias within each included RCT, and thus, we cannot speak to how the quality of each study may have contributed to variation and bias in study findings. Nevertheless, analyses of the risk for bias within RCTs evaluating the effect of periodontal treatment have been previously published.^112^, ^113^, ^114^, ^115^, ^116^, ^117^, ^118^ Second, this study is inherently limited by the search strategy and bibliographic databases used. However, we enlisted the help of a health sciences librarian and modeled our strategy after previously published research.^8^ Third, we were unable to systematically track potential confounding factors of PD beyond smoking status, including poor health behaviors, socioeconomic status, and health literacy levels. Finally, while we measured other potential sources of bias in our coding sheet (e.g., underrepresented races in study samples), we did not focus on these factors in our analysis. Nevertheless, these underlying population characteristics could also be contributing to our limited understanding of how findings could vary across specific populations.

## 5 CONCLUSION

Variation in study design characteristics may be contributing to mixed findings across RCTs investigating the effect of NSPT on systemic health, and ultimately, inconclusive findings within systematic reviews and meta‐analyses that combine results from these varied RCTs.

## AUTHOR CONTRIBUTIONS

Timothy Treat and Heather Taylor contributed to the (1) conception and design; (2) acquisition, analysis, and interpretation of data; and (3) drafted and critically revised the manuscript. Dylan Jones, Natalie Lorenzano, Andrew Bartels, and Scott Umberfield contributed to the (1) design; (2) acquisition, analysis, and interpretation of data; and (3) critically revised the manuscript. Titus Schleyer contributed to the (1) conception and design, (2) interpretation of data, and (3) critically revised the manuscript. All authors gave final approval and agreed to be accountable for all aspects of the work.

## CONFLICT OF INTEREST STATEMENT

The authors declared no potential conflicts of interest with respect to the research, authorship, and/or publication of this article.

## Supporting information



Supporting Information

## Data Availability

The data that support the findings of this study are available from the corresponding author upon reasonable request.

## References

[1] Eke PI , Borgnakke WS , Genco RJ . Recent epidemiologic trends in periodontitis in the USA. Periodontol 2000. 2020;82(1):257‐267. 31850640 10.1111/prd.12323

[2] Könönen E , Gursoy M , GursoyUK . Periodontitis: a multifaceted disease of tooth‐supporting tissues. J Clin Med Res. 2019;8(8):1135. doi:10.3390/jcm8081135 PMC6723779 31370168

[3] Naito M , Yuasa H , Nomura Y , Nakayama T , Hamajima N , Hanada N . Oral health status and health‐related quality of life: a systematic review. J Oral Sci. 2006;48(1):1‐7. 16617194 10.2334/josnusd.48.1

[4] Gil‐Montoya JA , de Mello ALF , Barrios R , Gonzalez‐Moles MA , Bravo M . Oral health in the elderly patient and its impact on general well‐being: a nonsystematic review. Clin Interv Aging. 2015;10:461‐467. 25709420 10.2147/CIA.S54630 PMC4334280

[5] Ortíz‐Barrios LB , Granados‐García V , Cruz‐Hervert P , Moreno‐Tamayo K , Heredia‐Ponce E , Sánchez‐García S . The impact of poor oral health on the oral health‐related quality of life (OHRQoL) in older adults: the oral health status through a latent class analysis. BMC Oral Health. 2019;19(1):141. doi:10.1186/s12903-019-0840-3 31291933 PMC6622000

[6] Bui FQ , Almeida‐da‐Silva CLC , Huynh B , et al. Association between periodontal pathogens and systemic disease. Biomed J. 2019;42(1):27‐35. 30987702 10.1016/j.bj.2018.12.001 PMC6468093

[7] Monsarrat P , Blaizot A , Kémoun P , et al. Clinical research activity in periodontal medicine: a systematic mapping of trial registers. J Clin Periodontol. 2016;43(5):390‐400. 26881700 10.1111/jcpe.12534

[8] Taylor HL , Rahurkar S , Treat TJ , Thyvalikakath TP , Schleyer TK . Does nonsurgical periodontal treatment improve systemic health?. J Dent Res. 2021;100(3):253‐260. 33089733 10.1177/0022034520965958 PMC7903843

[9] Carrizales‐Sepúlveda EF , Ordaz‐Farías A , Vera‐Pineda R , Flores‐Ramírez R . Periodontal disease, systemic inflammation and the risk of cardiovascular disease. Heart Lung Circ. 2018;27(11):1327‐1334. 29903685 10.1016/j.hlc.2018.05.102

[10] Faggion CM Jr , Cullinan MP , Atieh M . An overview of systematic reviews on the effectiveness of periodontal treatment to improve glycaemic control. J Periodontal Res. 2016;51(6):716‐725. 26913689 10.1111/jre.12358

[11] Shaqman M , Al‐Abedalla K , Wagner J , Swede H , Gunsolley JC , Ioannidou E . Reporting quality and spin in abstracts of randomized clinical trials of periodontal therapy and cardiovascular disease outcomes. PLoS One. 2020;15(4):e0230843. 32302309 10.1371/journal.pone.0230843 PMC7164582

[12] Sabharwal A , Gomes‐Filho IS , Stellrecht E , Scannapieco FA . Role of periodontal therapy in management of common complex systemic diseases and conditions: an update. Periodontol 2000. 2018;78(1):212‐226. 30198128 10.1111/prd.12226

[13] Liberati A , Altman DG , Tetzlaff J , et al. The PRISMA statement for reporting systematic reviews and meta‐analyses of studies that evaluate healthcare interventions: explanation and elaboration. BMJ. 2009;339:b2700. 19622552 10.1136/bmj.b2700 PMC2714672

[14] Sanz I , Alonso B , Carasol M , Herrera D , Sanz M . Nonsurgical treatment of periodontitis. J Evid Based Dent Pract. 2012;12(3 Suppl):76‐86. 23040340 10.1016/S1532-3382(12)70019-2

[15] Smiley CJ , Tracy SL , Abt E , et al. Evidence‐based clinical practice guideline on the nonsurgical treatment of chronic periodontitis by means of scaling and root planing with or without adjuncts. J Am Dent Assoc. 2015;146(7):525‐535. 26113100 10.1016/j.adaj.2015.01.026

[16] Aldridge JP , Lester V , Watts TL , Collins A , Viberti G , Wilson RF . Single‐blind studies of the effects of improved periodontal health on metabolic control in type 1 diabetes mellitus. J Clin Periodontol. 1995;22(4):271‐275. 7622632 10.1111/j.1600-051x.1995.tb00147.x

[17] Artese HPC , Longo PL , Gomes GH , Mayer MPA , Romito GA . Supragingival biofilm control and systemic inflammation in patients with type 2 diabetes mellitus. Braz Oral Res. 2015;29. doi:10.1590/1807-3107BOR-2015.vol29.0071 26039911

[18] Bian Y , Liu C , Fu Z . Application value of combination therapy of periodontal curettage and root planing on moderate‐to‐severe chronic periodontitis in patients with type 2 diabetes. Head Face Med. 2021;17(1):12. 33832490 10.1186/s13005-020-00253-z PMC8028763

[19] Chen L , Luo G , Xuan D , et al. Effects of non‐surgical periodontal treatment on clinical response, serum inflammatory parameters, and metabolic control in patients with type 2 diabetes: a randomized study. J Periodontol. 2012;83(4):435‐443. 21859323 10.1902/jop.2011.110327

[20] D'Aiuto F , Gkranias N , Bhowruth D , et al. Systemic effects of periodontitis treatment in patients with type 2 diabetes: a 12 month, single‐centre, investigator‐masked, randomised trial. Lancet Diabetes Endocrinol. 2018;6(12):954‐965. 30472992 10.1016/S2213-8587(18)30038-X

[21] El‐Makaky Y , Shalaby HK . The effects of non‐surgical periodontal therapy on glycemic control in diabetic patients: a randomized controlled trial. Oral Dis. 2020;26(4):822‐829. 31834660 10.1111/odi.13256

[22] Engebretson SP , Hyman LG , Michalowicz BS , et al. The effect of nonsurgical periodontal therapy on hemoglobin A1c levels in persons with type 2 diabetes and chronic periodontitis: a randomized clinical trial. JAMA. 2013;310(23):2523‐2532. 24346989 10.1001/jama.2013.282431 PMC4089989

[23] Gay IC , Tran DT , Cavender AC , et al. The effect of periodontal therapy on glycaemic control in a Hispanic population with type 2 diabetes: a randomized controlled trial. J Clin Periodontol. 2014;41(7):673‐680. 24797222 10.1111/jcpe.12268 PMC4080623

[24] Geisinger ML , Michalowicz BS , Hou W , et al. Systemic inflammatory biomarkers and their association with periodontal and diabetes‐related factors in the diabetes and periodontal therapy trial, a randomized controlled trial. J Periodontol. 2016;87(8):900‐913. 27108476 10.1902/jop.2016.150727

[25] Jones JA , Miller DR , Wehler CJ , et al. Does periodontal care improve glycemic control? The department of veterans affairs dental diabetes study. J Clin Periodontol. 2007;34(1):46‐52. 17137468 10.1111/j.1600-051X.2006.01002.x

[26] Kapellas K , Mejia G , Bartold PM , et al. Periodontal therapy and glycaemic control among individuals with type 2 diabetes: reflections from the PerioCardio study. Int J Dent Hyg. 2017;15(4):e42‐e51. 27245786 10.1111/idh.12234

[27] Kaur PK , Narula SC , Rajput R , K Sharma , R , Tewari S . Periodontal and glycemic effects of nonsurgical periodontal therapy in patients with type 2 diabetes stratified by baseline HbA1c. J Oral Sci. 2015;57(3):201‐211. 26369484 10.2334/josnusd.57.201

[28] Kiran M , Arpak N , Unsal E , Erdoğan MF . The effect of improved periodontal health on metabolic control in type 2 diabetes mellitus. J Clin Periodontol. 2005;32(3):266‐272. 15766369 10.1111/j.1600-051X.2005.00658.x

[29] Koromantzos PA , Makrilakis K , Dereka X , Katsilambros N , Vrotsos IA , Madianos PN . A randomized, controlled trial on the effect of non‐surgical periodontal therapy in patients with type 2 diabetes. Part I: effect on periodontal status and glycaemic control. J Clin Periodontol. 2011;38(2):142‐147. 10.1111/j.1600-051X.2010.01652.x 21114680

[30] Koromantzos PA , Makrilakis K , Dereka X , et al. Effect of non‐surgical periodontal therapy on C‐reactive protein, oxidative stress, and matrix metalloproteinase (MMP)‐9 and MMP‐2 levels in patients with type 2 diabetes: a randomized controlled study. J Periodontol. 2012;83(1):3‐10. 21627458 10.1902/jop.2011.110148

[31] Masi S , Orlandi M , Parkar M , et al. Mitochondrial oxidative stress, endothelial function and metabolic control in patients with type II diabetes and periodontitis: a randomised controlled clinical trial. Int J Cardiol. 2018;271:263‐268. 30077530 10.1016/j.ijcard.2018.05.019 PMC6152589

[32] Mauri‐Obradors E , Merlos A , Estrugo‐Devesa A , Jané‐Salas E , López‐López J , Viñas M . Benefits of non‐surgical periodontal treatment in patients with type 2 diabetes mellitus and chronic periodontitis: a randomized controlled trial. J Clin Periodontol. 2018;45(3):345‐353. 29265454 10.1111/jcpe.12858

[33] Mizuno H , Ekuni D , Maruyama T , et al. The effects of non‐surgical periodontal treatment on glycemic control, oxidative stress balance and quality of life in patients with type 2 diabetes: a randomized clinical trial. PLoS One. 2017;12(11):e0188171. 29145468 10.1371/journal.pone.0188171 PMC5689834

[34] Moeintaghavi A , Arab HR , Bozorgnia Y , Kianoush K , Alizadeh M . Non‐surgical periodontal therapy affects metabolic control in diabetics: a randomized controlled clinical trial. Aust Dent J. 2012;57(1):31‐37. 22369555 10.1111/j.1834-7819.2011.01652.x

[35] Pham TAV , Nguyen PA , Tran TTP , Nguyen VTT . Nonsurgical periodontal treatment improved the type 2 diabetes mellitus status in smokers: a randomized controlled trial. Diabetes Res Clin Pract. 2022;194:110150. 36375565 10.1016/j.diabres.2022.110150

[36] Nishioka S , Maruyama K , Tanigawa T , et al. Effect of non‐surgical periodontal therapy on insulin resistance and insulin sensitivity among individuals with borderline diabetes: a randomized controlled trial. J Dent. 2019;85:18‐24. 30986513 10.1016/j.jdent.2019.04.005

[37] Raman RPC , Taiyeb‐Ali TB , Chan SP , Chinna K , Vaithilingam RD . Effect of nonsurgical periodontal therapy verses oral hygiene instructions on type 2 diabetes subjects with chronic periodontitis: a randomised clinical trial. BMC Oral Health. 2014;14:79. 24965218 10.1186/1472-6831-14-79 PMC4082680

[38] Rapone B , Ferrara E , Corsalini M , et al. Inflammatory status and glycemic control level of patients with type 2 diabetes and periodontitis: a randomized clinical trial. Int J Environ Res Public Health. 2021;18(6):3018. doi:10.3390/ijerph18063018 33804123 PMC7998112

[39] Sun WL , Chen LL , Zhang SZ , Wu YM , Ren YZ , Qin GM . Inflammatory cytokines, adiponectin, insulin resistance and metabolic control after periodontal intervention in patients with type 2 diabetes and chronic periodontitis. Intern Med. 2011;50(15):1569‐1574. 21804283 10.2169/internalmedicine.50.5166

[40] Syed NK . Effects of nonsurgical periodontal therapy on glycemic control in diabetic patients under systemic administration of antidiabetic ayurvedic drug. J Contemp Dent Pract. 2023;24(7):481‐484. 37622627 10.5005/jp-journals-10024-3499

[41] Tran TT , Ngo QTT , Tran DH , Nguyen TDT . Effect of two nonsurgical periodontal treatment modalities in type 2 diabetes mellitus patients with chronic periodontitis: a randomized clinical trial. J Contemp Dent Pract. 2021;22(11):1275‐1280. 35343453

[42] Tsobgny‐Tsague NF , Lontchi‐Yimagou E , Nana ARN , et al. Effects of nonsurgical periodontal treatment on glycated haemoglobin on type 2 diabetes patients (PARODIA 1 study): a randomized controlled trial in a sub‐Saharan Africa population. BMC Oral Health. 2018;18(1):28. 29482543 10.1186/s12903-018-0479-5 PMC5828384

[43] Vergnes JN , Canceill T , Vinel A , et al. The effects of periodontal treatment on diabetic patients: the DIAPERIO randomized controlled trial. J Clin Periodontol. 2018;45(10):1150‐1163. 30136741 10.1111/jcpe.13003

[44] Wu Y , Chen L , Wei B , Luo K , Yan F . Effect of non‐surgical periodontal treatment on visfatin concentrations in serum and gingival crevicular fluid of patients with chronic periodontitis and type 2 diabetes mellitus. J Periodontol. 2015;86(6):795‐800. 25786566 10.1902/jop.2015.140476

[45] Yun F , Firkova EI , Jun‐Qi L , Xun H . Effect of non‐surgical periodontal therapy on patients with type 2 diabetes mellitus. Folia Med. 2007;49(1‐2):32‐36. 18018467

[46] Wang S , Liu J , Zhang J , et al. Glycemic control and adipokines after periodontal therapy in patients with Type 2 diabetes and chronic periodontitis. Braz Oral Res. 2017;31:e90. 29185604 10.1590/1807-3107BOR-2017.vol31.0090

[47] Wang Y , Liu HN , Zhen Z , et al. A randomized controlled trial of the effects of non‐surgical periodontal therapy on cardiac function assessed by echocardiography in type 2 diabetic patients. J Clin Periodontol. 2020;47(6):726‐736. 32350903 10.1111/jcpe.13291

[48] Caneiro‐Queija L , López‐Carral J , Martin‐Lancharro P , Limeres‐Posse J , Diz‐Dios P , Blanco‐Carrion J . Non‐surgical treatment of periodontal disease in a pregnant Caucasian women population: adverse pregnancy outcomes of a randomized clinical trial. Int J Environ Res Public Health. 2019;16(19):3638. doi:10.3390/ijerph16193638 31569780 PMC6801449

[49] Fiorini T , Susin C , da Rocha JM , et al. Effect of nonsurgical periodontal therapy on serum and gingival crevicular fluid cytokine levels during pregnancy and postpartum. J Periodontal Res. 2013;48(1):126‐133. 22835005 10.1111/j.1600-0765.2012.01513.x

[50] Jeffcoat MK , Hauth JC , Geurs NC , et al. Periodontal disease and preterm birth: results of a pilot intervention study. J Periodontol. 2003;74(8):1214‐1218. 14514236 10.1902/jop.2003.74.8.1214

[51] López NJ , Smith PC , Gutierrez J . Periodontal therapy may reduce the risk of preterm low birth weight in women with periodontal disease: a randomized controlled trial. J Periodontol. 2002;73(8):911‐924. 12211502 10.1902/jop.2002.73.8.911

[52] Macones GA , Parry S , Nelson DB , et al. Treatment of localized periodontal disease in pregnancy does not reduce the occurrence of preterm birth: results from the Periodontal Infections and Prematurity Study (PIPS). Am J Obstet Gynecol. 2010;202(2):147. e1‐8. 10.1016/j.ajog.2009.10.892 20113691

[53] Michalowicz BS , Novak MJ , Hodges JS , et al. Serum inflammatory mediators in pregnancy: changes after periodontal treatment and association with pregnancy outcomes. J Periodontol. 2009;80(11):1731‐1741. 19905943 10.1902/jop.2009.090236 PMC2922720

[54] Michalowicz BS , Hodges JS , DiAngelis AJ , et al. Treatment of periodontal disease and the risk of preterm birth. N Engl J Med. 2006;355(18):1885‐1894. 17079762 10.1056/NEJMoa062249

[55] Michalowicz BS , DiAngelis AJ , Novak MJ , et al. Examining the safety of dental treatment in pregnant women. J Am Dent Assoc. 2008;139(6):685‐695. 18519992 10.14219/jada.archive.2008.0250

[56] Newnham JP , Newnham IA , Ball CM , et al. Treatment of periodontal disease during pregnancy: a randomized controlled trial. Obstet Gynecol. 2009;114(6):1239‐1248. 19935025 10.1097/AOG.0b013e3181c15b40

[57] Offenbacher S , Beck JD , Jared HL , et al. Effects of periodontal therapy on rate of preterm delivery: a randomized controlled trial. Obstet Gynecol. 2009;114(3):551‐559. 19701034 10.1097/AOG.0b013e3181b1341f PMC2917914

[58] Offenbacher S , Beck J , Jared H , et al. 3: maternal oral therapy to reduce obstetric risk (MOTOR): a report of a multi‐centered periodontal therapy randomized‐controlled trial on rate of preterm delivery. Am J Obstet Gynecol. 2008;199(6):S2.

[59] Oliveira AMSD , de Oliveira PAD , Cota LOM , Magalhães CS , Moreira AN , Costa FO . Periodontal therapy and risk for adverse pregnancy outcomes. Clin Oral Investig. 2011;15(5):609‐615. 10.1007/s00784-010-0424-8 20495936

[60] Penova‐Veselinovic B , Keelan JA , Wang CA , Newnham JP , Pennell CE . Changes in inflammatory mediators in gingival crevicular fluid following periodontal disease treatment in pregnancy: relationship to adverse pregnancy outcome. J Reprod Immunol. 2015;112:1‐10. 26093363 10.1016/j.jri.2015.05.002

[61] Pirie M , Linden G , Irwin C . Intrapregnancy non‐surgical periodontal treatment and pregnancy outcome: a randomized controlled trial. J Periodontol. 2013;84(10):1391‐1400. 23237583 10.1902/jop.2012.120572

[62] Radnai M , Pál A , Novák T , Urbán E , Eller J , Gorzó I . Benefits of periodontal therapy when preterm birth threatens. J Dent Res. 2009;88(3):280‐284. 19329465 10.1177/0022034508330229

[63] Reddy BVR , Tanneeru S , Chava VK . The effect of phase‐I periodontal therapy on pregnancy outcome in chronic periodontitis patients. J Obstet Gynaecol. 2014;34(1):29‐32. 24359045 10.3109/01443615.2013.829029

[64] Sadatmansouri S , Sedighpoor N , Aghaloo M . Effects of periodontal treatment phase I on birth term and birth weight. J Indian Soc Pedod Prev Dent. 2006;24(1):23‐26. 16582527 10.4103/0970-4388.22831

[65] Tarannum F , Faizuddin M . Effect of periodontal therapy on pregnancy outcome in women affected by periodontitis. J Periodontol. 2007;78(11):2095‐2103. 17970675 10.1902/jop.2007.060388

[66] Weidlich P , Moreira CHC , Fiorini T , et al. Effect of nonsurgical periodontal therapy and strict plaque control on preterm/low birth weight: a randomized controlled clinical trial. Clin Oral Investig. 2013;17(1):37‐44. 10.1007/s00784-012-0679-3 22302453

[67] Beck JD , Couper DJ , Falkner KL , et al. The Periodontitis and Vascular Events (PAVE) pilot study: adverse events. J Periodontol. 2008;79(1):90‐96. 18166097 10.1902/jop.2008.070223

[68] Bokhari SAH , Khan AA , Butt AK , et al. Non‐surgical periodontal therapy reduces coronary heart disease risk markers: a randomized controlled trial. J Clin Periodontol. 2012;39(11):1065‐1074. 22966824 10.1111/j.1600-051X.2012.01942.x

[69] Caúla AL , Lira‐Junior R , Tinoco EMB , Fischer RG . The effect of periodontal therapy on cardiovascular risk markers: a 6‐month randomized clinical trial. J Clin Periodontol. 2014;41(9):875‐882. 25041550 10.1111/jcpe.12290

[70] Czesnikiewicz‐Guzik M , Osmenda G , Siedlinski M , et al. Causal association between periodontitis and hypertension: evidence from Mendelian randomization and a randomized controlled trial of non‐surgical periodontal therapy. Eur Heart J. 2019;40(42):3459‐3470. doi:10.1093/eurheartj/ehz646 31504461 PMC6837161

[71] Hada DS , Garg S , Ramteke GB , Ratre MS . Effect of non‐surgical periodontal treatment on clinical and biochemical risk markers of cardiovascular disease: a randomized trial. J Periodontol. 2015;86(11):1201‐1211. 26205747 10.1902/jop.2015.150249

[72] Ide M , McPartlin D , Coward PY , Crook M , Lumb P , Wilson RF . Effect of treatment of chronic periodontitis on levels of serum markers of acute‐phase inflammatory and vascular responses. J Clin Periodontol. 2003;30(4):334‐340. 12694432 10.1034/j.1600-051x.2003.00282.x

[73] Lobo MG , Schmidt MM , Lopes RD , et al. Treating periodontal disease in patients with myocardial infarction: a randomized clinical trial. Eur J Intern Med. 2020;71:76‐80. 31810741 10.1016/j.ejim.2019.08.012

[74] Montenegro MM , Ribeiro IWJ , Kampits C , et al. Randomized controlled trial of the effect of periodontal treatment on cardiovascular risk biomarkers in patients with stable coronary artery disease: preliminary findings of 3 months. J Clin Periodontol. 2019;46(3):321‐331. 30761568 10.1111/jcpe.13085

[75] Offenbacher S , Beck JD , Moss K , et al. Results from the Periodontitis and Vascular Events (PAVE) Study: a pilot multicentered, randomized, controlled trial to study effects of periodontal therapy in a secondary prevention model of cardiovascular disease. J Periodontol. 2009;80(2):190‐201. 19186958 10.1902/jop.2009.080007 PMC2778200

[76] Rapone B , Ferrara E , Qorri E , et al. The impact of periodontal inflammation on endothelial function assessed by circulating levels of asymmetric dimethylarginine: a single‐blinded randomized clinical trial. J Clin Med Res. 2022;11(14):4173. doi:10.3390/jcm11144173 PMC9316194 35887937

[77] Saffi MAL , Rabelo‐Silva ER , Polanczyk CA , et al. Periodontal therapy and endothelial function in coronary artery disease: a randomized controlled trial. Oral Dis. 2018;24(7):1349‐1357. 29873864 10.1111/odi.12909

[78] Seinost G , Horina A , Arefnia B , et al. Periodontal treatment and vascular inflammation in patients with advanced peripheral arterial disease: a randomized controlled trial. Atherosclerosis. 2020;313:60‐69. 33032234 10.1016/j.atherosclerosis.2020.09.019

[79] Sen S , Curtis J , Hicklin D , et al. Periodontal disease treatment after stroke or transient ischemic attack: the PREMIERS study, a randomized clinical trial. Stroke. 2023;54(9):2214‐2222. 37548008 10.1161/STROKEAHA.122.042047 PMC10668075

[80] Taylor B , Tofler G , Morel‐Kopp MC , et al. The effect of initial treatment of periodontitis on systemic markers of inflammation and cardiovascular risk: a randomized controlled trial. Eur J Oral Sci. 2010;118(4):350‐356. 20662907 10.1111/j.1600-0722.2010.00748.x

[81] Tonetti MS , D'Aiuto F , Nibali L , et al. Treatment of periodontitis and endothelial function. N Engl J Med. 2007;356(9):911‐920. 17329698 10.1056/NEJMoa063186

[82] Vidal F , Figueredo CMS , Cordovil I , Fischer RG . Periodontal therapy reduces plasma levels of interleukin‐6, C‐reactive protein, and fibrinogen in patients with severe periodontitis and refractory arterial hypertension. J Periodontol. 2009;80(5):786‐791. 19405832 10.1902/jop.2009.080471

[83] Zhou QB , Xia WH , Ren J , et al. Effect of intensive periodontal therapy on blood pressure and endothelial microparticles in patients with prehypertension and periodontitis: a randomized controlled trial. J Periodontol. 2017;88(8):711‐722. 28452620 10.1902/jop.2017.160447

[84] Isola G , Tartaglia GM , Santonocito S , Polizzi A , Williams RC , Iorio‐Siciliano V . Impact of N‐terminal pro‐B‐type natriuretic peptide and related inflammatory biomarkers on periodontal treatment outcomes in patients with periodontitis: an explorative human randomized‐controlled clinical trial. J Periodontol. 2023;94(12):1414‐1424. 37433155 10.1002/JPER.23-0063

[85] Fu YW , Li XX , Xu HZ , Gong YQ , Yang Y . Effects of periodontal therapy on serum lipid profile and proinflammatory cytokines in patients with hyperlipidemia: a randomized controlled trial. Clin Oral Investig. 2016;20(6):1263‐1269. 10.1007/s00784-015-1621-2 26434651

[86] Oz SG , Fentoglu O , Kilicarslan A , et al. Beneficial effects of periodontal treatment on metabolic control of hypercholesterolemia. South Med J. 2007;100(7):686‐691. 17639748 10.1097/SMJ.0b013e31802fa327

[87] Chung WC , Kao CC , Huang CF , Lee CY , Lu HK , Wu MS . Effects of periodontal treatment in patients with periodontitis and kidney failure: a pilot study. Int J Environ Res Public Health. 2022;19(3):1533. doi:10.3390/ijerph19031533 35162556 PMC8835327

[88] Fang F , Wu B , Qu Q , et al. The clinical response and systemic effects of non‐surgical periodontal therapy in end‐stage renal disease patients: a 6‐month randomized controlled clinical trial. J Clin Periodontol. 2015;42(6):537‐546. 25933364 10.1111/jcpe.12411

[89] Vachhani KS , Bhavsar NV . Effects of non‐surgical periodontal therapy on serum inflammatory factor high‐sensitive C‐reactive protein, periodontal parameters and renal biomarkers in patients with chronic periodontitis and chronic kidney disease. Dent Med Probl. 2021;58(4):489‐498. 34816635 10.17219/dmp/136034

[90] Wehmeyer MMH , Kshirsagar AV , Barros SP , et al. A randomized controlled trial of intensive periodontal therapy on metabolic and inflammatory markers in patients With ESRD: results of an exploratory study. Am J Kidney Dis. 2013;61(3):450‐458. 23261122 10.1053/j.ajkd.2012.10.021 PMC3578050

[91] Al‐Katma MK , Bissada NF , Bordeaux JM , Sue J , Askari AD . Control of periodontal infection reduces the severity of active rheumatoid arthritis. J Clin Rheumatol. 2007;13(3):134‐137. 17551378 10.1097/RHU.0b013e3180690616

[92] Nguyen VB , Nguyen TT , Huynh NCN , Nguyen KD , Le TA , Hoang HT . Effects of non‐surgical periodontal treatment in rheumatoid arthritis patients: a randomized clinical trial. Dent Med Probl. 2021;58(1):97‐105. 33792210 10.17219/dmp/131266

[93] Thilagar S , Theyagarajan R , Mugri MH , et al. Periodontal treatment for chronic periodontitis with rheumatoid arthritis. Int Dent J. 2022;72(6):832‐838. 35810012 10.1016/j.identj.2022.04.008 PMC9676424

[94] Basher SS , Saub R , Vaithilingam RD , et al. Impact of non‐surgical periodontal therapy on OHRQoL in an obese population, a randomised control trial. Health Qual Life Outcomes. 2017;15(1):225. 29157276 10.1186/s12955-017-0793-7 PMC5696769

[95] Milanesi FC , Greggianin BF , Dos Santos GO , et al. Effect of periodontal treatment on glycated haemoglobin and metabolic syndrome parameters: a randomized clinical trial. J Clin Periodontol. 2023;50(1):11‐21. 36053828 10.1111/jcpe.13717

[96] Montero E , López M , Vidal H , et al. Impact of periodontal therapy on systemic markers of inflammation in patients with metabolic syndrome: a randomized clinical trial. Diabetes Obes Metab. 2020;22(11):2120‐2132. 32613714 10.1111/dom.14131

[97] Maybodi FR , Bashiri H , Sezavar K , Owlia F . Effect of periodontal treatment on serum inflammatory parameters and disease activity in patients with systemic lupus erythematosus: a randomized controlled trial. J Indian Soc Periodontol. 2022;26(6):564‐569. 36582961 10.4103/jisp.jisp_607_21 PMC9793920

[98] Manau C , Echeverria A , Agueda A , Guerrero A , Echeverria JJ . Periodontal disease definition may determine the association between periodontitis and pregnancy outcomes. J Clin Periodontol. 2008;35(5):385‐397. 18341599 10.1111/j.1600-051X.2008.01222.x

[99] Ioannidou E , Shaqman M , Burleson J , Dongari‐Bagtzoglou A . Periodontitis case definition affects the association with renal function in kidney transplant recipients. Oral Dis. 2010;16(7):636‐642. 20412451 10.1111/j.1601-0825.2010.01665.x PMC2910134

[100] Kok MGM , de Ronde MWJ , Moerland PD , Ruijter JM , Creemers EE , Pinto‐Sietsma SJ . Small sample sizes in high‐throughput miRNA screens: a common pitfall for the identification of miRNA biomarkers. Biomol Detect Quantif. 2018;15:1‐5. 29276692 10.1016/j.bdq.2017.11.002 PMC5737945

[101] Pepe MS , Li CI , Feng Z . Improving the quality of biomarker discovery research: the right samples and enough of them. Cancer Epidemiol Biomarkers Prev. 2015;24(6):944‐950. 25837819 10.1158/1055-9965.EPI-14-1227 PMC4452419

[102] Caton JG , Armitage G , Berglundh T , et al. A new classification scheme for periodontal and peri‐implant diseases and conditions—introduction and key changes from the 1999 classification. J Clin Periodontol. 2018;45 Suppl 20:S1‐S8. 29926489 10.1111/jcpe.12935

[103] Burgan SW . The role of tobacco use in periodontal diseases: a literature review. Gen Dent. 1997;45(5):449‐460. 9515412

[104] Johnson GK , Slach NA . Impact of tobacco use on periodontal status. J Dent Educ. 2001;65(4):313‐321. 11336116

[105] Bergström J . Tobacco smoking and chronic destructive periodontal disease. Odontology. 2004;92(1):1‐8. 15490298 10.1007/s10266-004-0043-4

[106] Laxman VK , Annaji S . Tobacco use and its effects on the periodontium and periodontal therapy. J Contemp Dent Pract. 2008;9(7):97‐107. 18997922

[107] Leow NM , Moreno F , Marletta D , et al. Recurrence and progression of periodontitis and methods of management in long‐term care: a systematic review and meta‐analysis. J Clin Periodontol. 2022;49(Suppl 24):291‐313. 34761412 10.1111/jcpe.13553

[108] Clarkson JE , Pitts NB , Fee PA , et al. Examining the effectiveness of different dental recall strategies on maintenance of optimum oral health: the INTERVAL dental recalls randomised controlled trial. Br Dent J. 2021;230(4):236‐243. 33637927 10.1038/s41415-021-2612-0 PMC7908962

[109] Farooqi OA , Wehler CJ , Gibson G , Jurasic MM , Jones JA . Appropriate recall interval for periodontal maintenance: a systematic review. J Evid Based Dent Pract. 2015;15(4):171‐181. 26698003 10.1016/j.jebdp.2015.10.001 PMC4848042

[110] Riley P , Worthington HV , Clarkson JE , Beirne PV . Recall intervals for oral health in primary care patients. Cochrane Database Syst Rev. 2013;(12):CD004346. 24353242 10.1002/14651858.CD004346.pub4

[111] Patel S , Bay RC , Glick M . A systematic review of dental recall intervals and incidence of dental caries. J Am Dent Assoc. 2010;141(5):527‐539. 20436100 10.14219/jada.archive.2010.0225

[112] Iheozor‐Ejiofor Z , Middleton P , Esposito M , Glenny AM . Treating periodontal disease for preventing adverse birth outcomes in pregnant women. Cochrane Database Syst Rev. 2017;6:CD005297. 28605006 10.1002/14651858.CD005297.pub3 PMC6481493

[113] Kim AJ , Lo AJ , Pullin DA , Thornton‐Johnson DS , Karimbux NY . Scaling and root planing treatment for periodontitis to reduce preterm birth and low birth weight: a systematic review and meta‐analysis of randomized controlled trials. J Periodontol. 2012;83(12):1508‐1519. 22376207 10.1902/jop.2012.110636

[114] Liu W , Cao Y , Dong L , et al. Periodontal therapy for primary or secondary prevention of cardiovascular disease in people with periodontitis. Cochrane Database Syst Rev. 2019;12:CD009197.doi:10.1002/14651858.CD009197.pub4 31887786 PMC6953391

[115] D'Isidoro O , Perrotti V , Hui WL , Piattelli A , Iaculli F , Quaranta A . The impact of non‐surgical therapy of periodontal disease on surrogate markers for cardiovascular disease: a literature review. Am J Dent. 2019;32(4):191‐200. 31436940

[116] Roca‐Millan E , González‐Navarro B , Sabater‐Recolons MM , Marí‐Roig A , Jané‐Salas E , López‐López J . Periodontal treatment on patients with cardiovascular disease: systematic review and meta‐analysis. Med Oral Patol Oral Cir Bucal. 2018;23(6):e681‐e690. 30341272 10.4317/medoral.22725 PMC6261003

[117] Simpson TC , Weldon JC , Worthington HV , et al. Treatment of periodontal disease for glycaemic control in people with diabetes mellitus. Cochrane Database Syst Rev. 2015;(11):CD004714. 26545069 10.1002/14651858.CD004714.pub3 PMC6486035

[118] Baeza M , Morales A , Cisterna C , et al. Effect of periodontal treatment in patients with periodontitis and diabetes: systematic review and meta‐analysis. J Appl Oral Sci. 2020;28:e20190248. 31939522 10.1590/1678-7757-2019-0248 PMC6919200

