# Supplementary material for: Design characteristics of studies evaluating the effect of non‐surgical periodontal treatment on systemic health outcomes
Source: J Periodontol. 2025 Nov 19;97(4):619–32. doi: 10.1002/JPER.24-0847 (PMC13169483; doi:10.1002/JPER.24-0847)
Supplement: Supplementary file 1 — Supporting Information [file JPER-97-619-s001.docx]

**Supplementary Materials for manuscript entitled:** Design characteristics of studies evaluating the effect of non-surgical periodontal treatment on systemic health outcomes

**Authors:** Timothy Treat, DDS, Dylan Jones, BS, Natalie Lorenzano, DDS, Scott Umberfield, Andrew Bartels, DDS; Titus Schleyer, PhD, DMD; Heather Taylor, PhD, MPH, LDH

**Supplemental Table 1 – Search strategy for Embase**

| Search order | Search terms |
| --- | --- |
| 1 | 'cardiovascular disease'/exp OR 'cardiovascular disease' |
| 2 | 'cerebrovascular disease'/exp |
| 3 | 'rheumatoid arthritis'/exp |
| 4 | 'rheumatic disease'/exp |
| 5 | 'obesity'/exp |
| 6 | 'kidney disease'/exp |
| 7 | 'kidney failure'/exp |
| 8 | 'diabetes mellitus'/exp |
| 9 | 'metabolic disorder'/exp |
| 10 | 'pregnancy'/exp |
| 11 | 'pregnancy complication'/exp |
| 12 | 'periodontal disease'/exp |
| 13 | 'periodontitis'/exp |
| 14 | 'periodontics'/exp |
| 15 | 'preventive dentistry'/exp |
| 16 | 'mouth hygiene'/exp |
| 17 | 'dental prophylaxis'/exp |
| 18 | #1 OR #2 OR #3 OR #4 OR #5 OR #6 OR #7 OR #8 OR #9 OR #10 OR #11 |
| 19 | #12 OR #13 OR #14 OR #15 OR #16 OR #17 |
| 20 | #18 AND #19 |
| 21 | ('crossover procedure':de OR 'double-blind procedure':de OR 'randomized controlled trial':de OR 'single-blind procedure':de OR random*:de,ab,ti OR factorial*:de,ab,ti OR crossover*:de,ab,ti OR ((cross NEXT/1 over*):de,ab,ti) OR placebo*:de,ab,ti OR ((doubl* NEAR/1 blind*):de,ab,ti) OR ((singl* NEAR/1 blind*):de,ab,ti) OR assign*:de,ab,ti OR allocat*:de,ab,ti OR volunteer*:de,ab,ti) NOT ([animals]/lim NOT [humans]/lim) |
| 22 | #20 AND #21 |

**Supplemental Table 2 – Search strategy for Medline via Ovid**

| Search order | Search terms |
| --- | --- |
| 1 | exp cardiovascular disease/ |
| 2 | exp cerebrovascular disease/ |
| 3 | exp Atherosclerosis/ |
| 4 | exp Thrombosis/ |
| 5 | exp Pregnancy/ |
| 6 | exp Pregnancy complications/ |
| 7 | exp Diabetes Mellitus/ |
| 8 | exp Metabolic Diseases/ |
| 9 | exp Rheumatoid arthritis/ |
| 10 | exp Obesity/ |
| 11 | exp Kidney failure/ |
| 12 | exp Kidney Diseases/ |
| 13 | 1 or 2 or 3 or 4 or 5 or 6 or 7 or 8 or 9 or 10 or 11 or 12 |
| 14 | exp Periodontics/ |
| 15 | exp Periodontal diseases/ |
| 16 | exp Periodontitis/ |
| 17 | exp Preventive Dentistry/ |
| 18 | exp Oral Hygiene/ |
| 19 | exp Dental Prophylaxis/ |
| 20 | 14 or 15 or 16 or 17 or 18 or 19 |
| 21 | ((Randomized controlled trial.pt. or controlled clinical trial.pt. or randomized.ab. or placebo.ab. or drug therapy.fs. or randomly.ab. or trial.ab. or groups.ab.) not (exp animals/not humans.sh.)) |
| 22 | 13 and 20 and 21 |

**Supplemental Table 3 - List of excluded studies following full-text review and reasons for exclusion**

| **First author’s last name**  **(Study year)** | **Reason for exclusion** | **Detail** |
| --- | --- | --- |
| Ahmad (2018)^1^ | Ineligible Study Design | Nonrandomized study design |
| AlAmri (2016)^2^ | Ineligible Study Design | Nonrandomized study design |
| Altamash (2016) ^3^ | Ineligible Study Design | Nonrandomized study design |
| Alshehri (2015)^4^ | Ineligible Comparator | Control group received some form of SRP |
| Arefnia (2022)^5^ | Ineligible Study Design | Not a peer-reviewed paper – Poster presentation |
| Bokhari (2013)^6^ | Conference Proceeding | Published Conference Abstract |
| Botero (2013)^7^ | Ineligible Intervention | Intervention included pharmaceutical |
| Bukleta (2018)^8^ | Ineligible Study Design | Preliminary study report |
| Chandni (2015)^9^ | Conference Proceeding | Published Conference Abstract |
| Chapple (2014)^10^ | Ineligible Study Design | Commentary/Letter/Opinion Piece |
| D’Aiuto (2006)^11^ | Ineligible Comparator | Control group received some form of SRP |
| da Silva Júnior (2022)^12^ | Ineligible Intervention | Intervention included laser treatment |
| dePablo (2023)^13^ | Ineligible Study Design | Primary objective was to test the feasibility of an RCT study |
| Gao (2016)^14^ | Ineligible Language | Non-English |
| Gomes-Filho (2010)^15^ | Ineligible Study Design | Nonrandomized study design. |
| Grossi (1997)^16^ | Ineligible Intervention | All groups received treatment, but they got different antimicrobial regimens |
| Grubbs (2018)^17^ | Conference Proceeding | Published Conference Abstract |
| Grubbs (2020)^18^ | Ineligible Comparator | Control group received some form of SRP |
| Gugnani (2021)^19^ | Ineligible Study Design | Commentary/Letter/Opinion Piece |
| Gunupati (2011)^20^ | Ineligible Study Design | Case-control study design |
| Han(2010)^21^ | Ineligible language | Non-English |
| Herrera (2009)^22^ | Ineligible Study Design | Not all subjects had periodontal disease |
| Izuora (2015)^23^ | Ineligible Study Design | Nonrandomized study design |
| Jiang (2013)^24^ | Ineligible Study Design | Protocol description of an RCT |
| Joseph (2016)^25^ | Conference Proceeding | Published Conference Abstract |
| Joseph (2017)^26^ | Ineligible Study Design | Nonrandomized study design |
| Kaneko (2021)^27^ | Ineligible Study Design | Nonrandomized study design |
| Kapellas (2014)^28^ | Conference Proceeding | Published Conference Abstract |
| Karapesta (2022)^29^ | Conference Proceeding | Published Conference Abstract |
| Kothiwale (2013)^30^ | Conference Proceeding | Published Conference Abstract |
| Li (2011)^31^ | Ineligible Language | Non-English |
| Lobo (2016)^32^ | Conference Proceeding | Published Conference Abstract |
| Lu (2020)^33^ | Ineligible language | Non-English |
| Madden (2008)^34^ | Ineligible Comparator | Control group received some form of SRP |
| Maheshwari (2023)^35^ | Ineligible Study Design | Nonrandomized study design |
| Mammen (2017)^36^ | Ineligible Study Design | Nonrandomized study design |
| Mansouri (2006)^37^ | Ineligible language | Non-English |
| Marhl (2022)^38^ | Ineligible Intervention | Quadrant scaling |
| Mariotti (2013)^39^ | Ineligible Study Design | Nonrandomized study design |
| Matern (2020)^40^ | Ineligible Study Design | Nonrandomized study design |
| Matsumoto (2009)^41^ | Ineligible Intervention | Intervention included minocycline |
| Moller (2020)^42^ | Ineligible Study Design | Commentary/Letter/Opinion Piece |
| Novak (2008)^43^ | Ineligible Outcome | Examined periodontal outcomes |
| Novak (2018)^44^ | Ineligible Language | Non-English |
| O’Connell (2008)^45^ | Ineligible Comparator | Control group received some form of SRP |
| Offenbacher (2010)^46^ | Conference Proceeding | Published Conference Abstract |
| Ortiz (2009)^47^ | Ineligible Intervention | Intervention included tumor necrosis factor inhibitors |
| Padial- Molina (2023)^48^ | Ineligible Study Design | Intervention group received periodontal surgery |
| Penova-Veselinovic (2014)^49^ | Conference Proceeding | Published Conference Abstract |
| Phetnin N. 2020)^50^ | Conference Proceeding | Published Conference Abstract |
| Promsudthi (2005)^51^ | Ineligible Intervention | Intervention included doxycycline |
| Pulivarthi (2022)^52^ | Ineligible Intervention | Intervention included laser treatment |
| Quintero (2018)^53^ | Ineligible comparator | Control group received some form of SRP |
| Redd (2019)^54^ | Ineligible Study Design | RCT Protocol |
| Rodrigues (2003)^55^ | Ineligible Study Design | Control group received some form of SRP |
| Sant’Ana (2011)^56^ | Ineligible Study Design | Nonrandomized study design. |
| Santos (2013)^57^ | Ineligible Intervention | Intervention included chlorhexidine |
| Shelswell (2021)^58^ | Ineligible Study Design | Commentary/Letter/Opinion Piece |
| Simpson (2022)^59^ | Ineligible Study Design | Systematic Review Study Design |
| Skilton (2011)^60^ | Ineligible Study Design | Protocol description of an RCT |
| Stewart (2001)^61^ | Ineligible Study Design | Nonrandomized study design. |
| Sutherland (2017)^62^ | Conference Proceeding | Published Conference Abstract |
| Tao (2017)^63^ | Ineligible Study Design | Published abstract from a scientific conference |
| Ushida (2008)^64^ | Ineligible Intervention | Examined full mouth scaling compared to quadrant/partial mouth scaling |
| Valentine (2022)^65^ | Ineligible Study Design | A sub-analysis of RCT data |
| Walling (2010)^66^ | Ineligible Study Design | Review of an RCT |
| Zhou (2013)^67^ | Ineligible Study Design | Nonrandomized study design. |

*References for Supplemental Table 3:*

1. Ahmad A, Nazar Z, Swaminathan D. C-reactive protein levels and periodontal diseases during pregnancy in Malaysian women. *Oral Health Prev Dent*. 2018;16(3):281-289.

2. Al Amri MD, Kellesarian SV, Al-Kheraif AA, Malmstrom H, Javed F, Romanos GE. Effect of oral hygiene maintenance on HbA1c levels and peri-implant parameters around immediately-loaded dental implants placed in type-2 diabetic patients: 2 years follow-up. *Clin Oral Implants Res*. 2016;27(11):1439-1443.

3. Altamash M, Klinge B, Engstrom PE. Periodontal treatment and HbA1c levels in subjects with diabetes mellitus. *J Oral Rehabil*. 2016;43(1):31-38.

4. Alshehri FA, Javed F. Impact of scaling and root planing on clinical periodontal status and glycemic levels in prediabetic patients. *Interv Med Appl Sci*. 2015;7(1):17-21.

5. Arefnia B, Kulnik R, Horina A, Seinost G, Lindner M, Wimmer G. Effects of peridontal treatment on 18FDG PET/CT scans in patients with peripheral arterial diseases. *J Clin Periodontol*. 2022;49((Arefnia B.; Lindner M.; Wimmer G.) Department of Dental Medicine and Oral Health, Medical University of Graz, Graz, Austria(Kulnik R.) Department of Radiology, Division of Nuclear Medicine, Medical University of Graz, Graz, Austria(Horina A.; Seinost G.)):93.

6. Bokhari SA, Khan AA, Butt AK, et al. Non-surgical periodontal therapy reduces coronary heart disease risk markers: A randomized controlled trial. *Clin Trials*. 2013;10:S47.

7. Botero JE, Yepes FL, Ochoa SP, et al. Effects of periodontal non-surgical therapy plus azithromycin on glycemic control in patients with diabetes: a randomized clinical trial. *J Periodontal Res*. 2013;48(6):706-712.

8. Bukleta D, Krasniqi S, Beretta G, et al. Impact of combined non-surgical and surgical periodontal treatment in patients with type 2 diabetes mellitus-a preliminary report randomized clinical study. *Biomed Res*. 2018;29(3). doi:10.4066/biomedicalresearch.29-17-644

9. Chandni R, Mammen J, Joseraj MG, Joseph R. Effect of nonsurgical periodontal therapy on insulin resistance in patients with type 2 diabetes mellitus and chronic periodontitis. *Diabetes*. 2015;64:A692.

10. Chapple ILC, Borgnakke WS, Genco RJ. Hemoglobin A1c levels among patients with diabetes receiving nonsurgical periodontal treatment [2]. *JAMA - Journal of the American Medical Association*. 2014;311(18):1919-1920.

11. D’Aiuto F, Parkar M, Nibali L, Suvan J, Lessem J, Tonetti MS. Periodontal infections cause changes in traditional and novel cardiovascular risk factors: results from a randomized controlled clinical trial. *Am Heart J*. 2006;151(5):977-984.

12. da Silva Júnior FL, de Araújo Silva DN, da Silva Azevedo ML, et al. Efficacy of ILIB on periodontal clinical parameters and glycemic control in patients with periodontitis and type II diabetes—randomized clinical trial. *Lasers Med Sci*. 2022;37(3):1945-1952.

13. de Pablo P, Serban S, Lopez-Oliva I, et al. Outcomes of periodontal therapy in rheumatoid arthritis: The OPERA feasibility randomized trial. *J Clin Periodontol*. 2023;50(3):295-306.

14. Gao L, Sun XJ, Xie H, Nan SH, Xie HX. Effects of essential periodontal treatment on serum level of sCD40L and periodontal clinical parameters in patients with moderate to severe periodontitis at high risk of stroke. *Shanghai Kou Qiang Yi Xue*. 2016;25(5):574-578.

15. Gomes-Filho IS, Cruz SS, Costa Mda C, et al. Periodontal therapy and low birth weight: preliminary results from an alternative methodologic strategy. *J Periodontol*. 2010;81(12):1725-1733.

16. Grossi SG, Skrepcinski FB, DeCaro T, et al. Treatment of periodontal disease in diabetics reduces glycated hemoglobin. *J Periodontol*. 1997;68(8):713-719.

17. Grubbs V, Vittinghoff E, Garcia FM, Powe NR. Effect of periodontal therapy on CKD: Findings of the kidney and periodontal disease (KAPD) pilot randomized controlled trial. *J Am Soc Nephrol*. 2018;29((Grubbs V.) San Francisco General Hospital Renal Center, San Francisco, CA, United States(Grubbs V.) UCSF, San Francisco, CA, United States(Vittinghoff E.; Garcia F.M.) University of California, San Francisco, CA, United States(Powe N.R.) Priscilla Chan a):466-467.

18. Grubbs V, Garcia F, Vittinghoff E, et al. Nonsurgical periodontal therapy in CKD: Findings of the Kidney and Periodontal Disease (KAPD) pilot randomized controlled trial. *Kidney Med*. 2020;2(1):49-58.

19. Gugnani N, Gugnani S. Can treatment of severe periodontitis in patients with ST-segment elevation myocardial infarction improve endothelial function? *Evid Based Dent*. 2021;22(1):5-7.

20. Gunupati S, Chava VK, Krishna BP. Effect of phase i periodontal therapy on anti-cardiolipin antibodies in patients with acute myocardial infarction associated with chronic periodontitis. *J Periodontol*. 2011;82(12):1657-1664.

21. Han B, You L. [Effect of initial periodontal therapy on diabetic patients with chronic periodontitis]. *Zhonghua Kou Qiang Yi Xue Za Zhi*. 2010;45(5):282-286.

22. Herrera JA, Vélez-Medina S, Molano RM, et al. Periodontal intervention effects on pregnancy outcomes in women with preeclampsia. *Colomb Med*. 2009;40(2):177-184.

23. Izuora KE, Ezeanolue E, Neubauer M, et al. Changes in inflammation and bone turnover markers after treatment for periodontal disease in patients with diabetes. *Diabetes*. 2015;64:A129-A130.

24. Jiang H, Xiong X, Su Y, et al. A randomized controlled trial of pre-conception treatment for periodontal disease to improve periodontal status during pregnancy and birth outcomes. *BMC Pregnancy Childbirth*. 2013;13. doi:10.1186/1471-2393-13-228

25. Joseph R, Radhakrishnan C, Sasikumar M, Raj MGJ. Effect of nonsurgical periodontal therapy on glycosylated hemoglobin levels in prediabetic patients with chronic perio dontitis. *Diabetes*. 2016;65:A382.

26. Joseph R, Sasikumar M, Mammen J, Joseraj MG, Radhakrishnan C. Nonsurgical periodontal-therapy improves glycosylated hemoglobin levels in pre-diabetic patients with chronic periodontitis. *World J Diabetes*. 2017;8(5):213-221.

27. Kaneko C, Kobayashi T, Ito S, et al. Association among periodontitis severity, anti-agalactosyl immunoglobulin G titer, and the disease activity of rheumatoid arthritis. *J Periodontal Res*. 2021;56(4):702-709.

28. Kapellas K, Maple-Brown LJ, Bartold PM, et al. Effect of a periodontal intervention on pulse wave velocity in Indigenous Australians with periodontal disease: The PerioCardio randomized controlled trial. *Glob Heart*. 2014;9(1):e44.

29. Karapetsa D, Gennai S, Discepoli N, Marruganti C, Nisi M, Graziani F. Treatment of periodontits enhancea the treatment of rheumatoid arthritis: A randomized clinical trial. *J Clin Periodontol*. 2022;49((Karapetsa D.; Gennai S.; Marruganti C.; Nisi M.; Graziani F.) Department of Surgical,Medical and Molecular Pathology and Critical Care Medicine, Sub-Unit of Periodontology, Halitosis and Periodontal Medicine, University of Pisa, Pisa, Italy(Discepoli N.;):33.

30. Kothiwale SV, Kothiwale VA, Bhargava PV. Effect of non-invasive periodontal therapy on glycaemic control in type 2 diabetes mellitus patients - A randomized control trial. *Diabetes*. 2013;62:A229.

31. Li Z, Sha YQ, Zhang BX, Zhu L, Kang J. [Effect of community periodontal care intervention on periodontal health and glycemic control in type 2 diabetic patients with chronic periodontitis]. *Beijing Da Xue Xue Bao*. 2011;43(2):285-289.

32. Lobo MG, Dipp T, Petry I, et al. Treating periodontal disease in patients with ST-elevation myocardial infarction: A randomized clinical trial. *J Am Coll Cardiol*. 2016;68(18):B65.

33. Lu WL, Zhang XY, Liao Y, Chen HW, Liu YL, Song ZC. Clinical effect of periodontal initial therapy on peritoneal dialysis patients with periodontitis. *J Shanghai Jiatong Univ*. 2020;40(11):1489-1494.

34. Madden TE, Herriges B, Boyd LD, Laughlin G, Chiodo G, Rosenstein D. Alterations in HbA1c following minimal or enhanced non-surgical, non-antibiotic treatment of gingivitis or mild periodontitis in type 2 diabetic patients: a pilot trial. *J Contemp Dent Pract*. 2008;9(5):9-16.

35. Maheshwari S, Chahal GS, Grover V, et al. Impact of periodontal treatment on inflammatory oxidative stress in chronic kidney disease subjects: An interventional clinical trial. *Am J Dent*. 2023;36(1):15-20.

36. Mammen J, Vadakkekuttical RJ, George JM, Kaziyarakath JA, Radhakrishnan C. Effect of non-surgical periodontal therapy on insulin resistance in patients with type II diabetes mellitus and chronic periodontitis, as assessed by C-peptide and the Homeostasis Assessment Index. *J Investig Clin Dent*. 2017;8(3). doi:10.1111/jicd.12221

37. Mansouri SS, Esteghamati A, Yousefi Y. Evaluation of first phase non-surgical periodontal therapy on diabetes control. *Iranian Journal of Diabetes and Lipid Disorders*. 2006;6(1):E13.

38. Marhl U, Gennai S, Peric M, et al. Acute systemic inflammation in obese patients following full-mouth versus quadrant non-surgical treatment of periodontitis. *J Clin Periodontol*. 2022;49((Marhl U.; Gennai S.; Peric M.; Marruganti C.; Graziani F.) Department of Surgical, Medical and Molecular Pathology and Critical Care Medicine, University of Pisa, Pisa, Italy(Marhl U.; Gennai S.; Peric M.; Marruganti C.; Graziani F.) Sub-Unit of Periodon):23.

39. Mariotti G, Quaranta A, Merli M, Holtzman LP, Piemontese M. Chronic Periodontitis and Cardiovascular Disease: A Controlled Clinical Trial. *Eur J Inflam*. 2013;11(2):459-467.

40. Matern J, Koch R, Petersmann A, et al. Effect of periodontal therapy on adipokine biomarkers in overweight. *J Clin Periodontol*. 2020;47(7):842-850.

41. Matsumoto S, Ogawa H, Soda S, et al. Effect of antimicrobial periodontal treatment and maintenance on serum adiponectin in type 2 diabetes mellitus. *J Clin Periodontol*. 2009;36(2):142-148.

42. Moller B, Bender P, Eick S, et al. Treatment of severe periodontitis may improve clinical disease activity in otherwise treatment-refractory rheumatoid arthritis patients. *Rheumatology* . 2020;59(1):243-245.

43. Novak MJ, Novak KF, Hodges JS, et al. Periodontal bacterial profiles in pregnant women: Response to treatment and associations with birth outcomes in the obstetrics and periodontal therapy (OPT) study. *J Periodontol*. 2008;79(10):1870-1879.

44. Novak T, Radnai M, Kozinszky Z, et al. [Effect of the treatment of periodontal disease on the outcome of pregnancy]. *Orv Hetil*. 2018;159(24):978-984.

45. O’Connell PAA, Taba M, Nomizo A, et al. Effects of periodontal therapy on glycemic control and inflammatory markers. *J Periodontol*. 2008;79(5):774-783.

46. Offenbacher S, Beck JD, Jared HL, et al. Effects of periodontal therapy on rate of preterm delivery: A randomized controlled trial. *Obstet Gynecol Surv*. 2010;65(1):6-7.

47. Ortiz P, Bissada NF, Palomo L, et al. Periodontal therapy reduces the severity of active rheumatoid arthritis in patients treated with or without tumor necrosis factor inhibitors. *J Periodontol*. 2009;80(4):535-540.

48. Padial-Molina M, Gonzalez-Perez G, Martin-Morales N, Sanchez-Fernandez E, O’Valle F, Galindo-Moreno P. Periostin in the relation between periodontal disease and atherosclerotic coronary artery disease: A pilot randomized clinical study. *J Periodontal Res*. Published online December 22, 2023. doi:10.1111/jre.13229

49. Penova-Veselinovic B, Keelan J, Newnham JP, Pennell CE. Changes in inflammatory mediators in gingival crevicular fluid following periodontal disease treatment in pregnant women. *J Paediatr Child Health*. 2014;50:25-26.

50. Phetnin N. Effectiveness of the diabetic and oral care program for SENIOR (DOCS) in older people with type 2 diabetes mellitus (T2DM) in Muang district, NAKHON Ratchasima Province, Thailand. *Eur Geriatr Med*. 2020;11(SUPPL 1):S182-S183.

51. Promsudthi A, Pimapansri S, Deerochanawong C, Kanchanavasita W. The effect of periodontal therapy on uncontrolled type 2 diabetes mellitus in older subjects. *Oral Dis*. 2005;11(5):293-298.

52. Pulivarthi P, Chava V, Gunupati S. Salivary tumor necrosis factor-alpha levels in periodontitis associated with diabetes mellitus after low level laser therapy as an adjunct to scaling and root planning: A randomized clinical trial. *J Indian Soc Periodontol*. 2022;26(3):236-244.

53. Quintero AJ, Chaparro A, Quirynen M, et al. Effect of two periodontal treatment modalities in patients with uncontrolled type 2 diabetes mellitus: A randomized clinical trial. *J Clin Periodontol*. 2018;45(9):1098-1106.

54. Redd KT, Phillips ST, McMillian B, et al. PeRiodontal Treatment to Eliminate Minority Inequality and Rural Disparities in Stroke (PREMIERS): A Multicenter, Randomized, Controlled Study. *Int J Cerebrovasc Dis Stroke*. 2019;2(2). https://www.ncbi.nlm.nih.gov/pubmed/32159164

55. Rodrigues DC, Taba MJ, Novaes ABJ, Souza SLS, Grisi MFM. Effect of non-surgical periodontal therapy on glycemic control in patients with type 2 diabetes mellitus. *J Periodontol*. 2003;74(9):1361-1367.

56. Sant’Ana AC, Campos MR, Passanezi SC, Rezende ML, Greghi SL, Passanezi E. Periodontal treatment during pregnancy decreases the rate of adverse pregnancy outcome: a controlled clinical trial. *J Appl Oral Sci*. 2011;19(2):130-136.

57. Santos VR, Lima JA, Miranda TS, et al. Full-mouth disinfection as a therapeutic protocol for type-2 diabetic subjects with chronic periodontitis: twelve-month clinical outcomes: a randomized controlled clinical trial. *J Clin Periodontol*. 2013;40(2):155-162.

58. Shelswell J. Does periodontal treatment have an impact on metabolic control and systemic inflammation in patients with type 2 diabetes? *Evid Based Dent*. 2021;22(1):40-41.

59. Simpson TC, Clarkson JE, Worthington HV, et al. Treatment of periodontitis for glycaemic control in people with diabetes mellitus. *Cochrane Database Syst Rev*. 2022;2022(4). doi:10.1002/14651858.CD004714.pub4

60. Skilton MR, Maple-Brown LJ, Kapellas K, et al. The effect of a periodontal intervention on cardiovascular risk markers in Indigenous Australians with periodontal disease: the PerioCardio study. *BMC Public Health*. 2011;11:729.

61. Stewart JE, Wager KA, Friedlander AH, Zadeh HH. The effect of periodontal treatment on glycemic control in patients with type 2 diabetes mellitus. *J Clin Periodontol*. 2001;28(4):306-310.

62. Sutherland MW, Liu J, Pitiphat W, Dasanayake A, Merchant AT. The effect of periodontal treatment on preterm birth among pregnant women with periodontal disease: Utilizing inverse probability weighting to control for selection bias in a randomized controlled trial. *Ann Epidemiol*. 2017;27(8):532.

63. Tao J, Yang J, Zhou Q, et al. Efficacy of intensive periodontal therapy in primary prevention of dental hypertension development: A randomized controlled clinical trial. *Circulation*. 2017;136. https://www.embase.com/search/results?subaction=viewrecord&id=L619984434&from=export

64. Ushida Y, Koshy G, Kawashima Y, et al. Changes in serum interleukin-6, C-reactive protein and thrombomodulin levels under periodontal ultrasonic debridement. *J Clin Periodontol*. 2008;35(11):969-975.

65. Valentine GC, Perez K, Tsegaye AT, et al. Non-Surgical Periodontal Treatment During Pregnancy and Rates of Preterm Birth and Low Birthweight Neonates. *Am J Obstet Gynecol*. 2022;226(1):S181.

66. Walling AD. Does treating periodontal disease during pregnancy reduce preterm birth? *Am Fam Physician*. 2010;82(6):693.

67. Zhou SY, Duan XQ, Hu R, Ouyang XY. Effect of non-surgical periodontal therapy on serum levels of TNF-a, IL-6 and C-reactive protein in periodontitis subjects with stable coronary heart disease. *Chin J Dent Res*. 2013;16(2):145-151.

**Supplemental Table 4 – List of studies that met inclusion criteria**

1. Aldridge JP, Lester V, Watts TL, Collins A, Viberti G, Wilson RF. Single-blind studies of the effects of improved periodontal health on metabolic control in type 1 diabetes mellitus. *J Clin Periodontol*. 1995;22(4):271-275.
2. Artese HPC, Longo PL, Gomes GH, Mayer MPA, Romito GA. Supragingival biofilm control and systemic inflammation in patients with type 2 diabetes mellitus. *Braz Oral Res*. 2015;29. doi:10.1590/1807-3107BOR-2015.vol29.0071
3. Bian Y, Liu C, Fu Z. Application value of combination therapy of periodontal curettage and root planing on moderate-to-severe chronic periodontitis in patients with type 2 diabetes. *Head Face Med*. 2021;17(1):12.
4. Chen L, Luo G, Xuan D, et al. Effects of non-surgical periodontal treatment on clinical response, serum inflammatory parameters, and metabolic control in patients with type 2 diabetes: a randomized study. *J Periodontol*. 2012;83(4):435-443.
5. D’Aiuto F, Gkranias N, Bhowruth D, et al. Systemic effects of periodontitis treatment in patients with type 2 diabetes: a 12 month, single-centre, investigator-masked, randomised trial. *Lancet Diabetes Endocrinol*. 2018;6(12):954-965.
6. El-Makaky Y, Shalaby HK. The effects of non-surgical periodontal therapy on glycemic control in diabetic patients: A randomized controlled trial. *Oral Dis*. 2020;26(4):822-829.
7. Engebretson SP, Hyman LG, Michalowicz BS, et al. The effect of nonsurgical periodontal therapy on hemoglobin A1c levels in persons with type 2 diabetes and chronic periodontitis: a randomized clinical trial. *JAMA*. 2013;310(23):2523-2532.
8. Gay IC, Tran DT, Cavender AC, et al. The effect of periodontal therapy on glycaemic control in a Hispanic population with type 2 diabetes: a randomized controlled trial. *J Clin Periodontol*. 2014;41(7):673-680.
9. Geisinger ML, Michalowicz BS, Hou W, et al. Systemic inflammatory biomarkers and their association with periodontal and diabetes-related factors in the Diabetes and Periodontal Therapy Trial, A randomized controlled trial. *J Periodontol*. 2016;87(8):900-913.
10. Jones JA, Miller DR, Wehler CJ, et al. Does periodontal care improve glycemic control? The Department of Veterans Affairs Dental Diabetes Study. *J Clin Periodontol*. 2007;34(1):46-52.
11. Kapellas K, Mejia G, Bartold PM, et al. Periodontal therapy and glycaemic control among individuals with type 2 diabetes: reflections from the PerioCardio study. *Int J Dent Hyg*. 2017;15(4):e42-e51.
12. Kaur PK, Narula SC, Rajput R, K Sharma R, Tewari S. Periodontal and glycemic effects of nonsurgical periodontal therapy in patients with type 2 diabetes stratified by baseline HbA1c. *J Oral Sci*. 2015;57(3):201-211.
13. Kiran M, Arpak N, Unsal E, Erdoğan MF. The effect of improved periodontal health on metabolic control in type 2 diabetes mellitus. *J Clin Periodontol*. 2005;32(3):266-272.
14. Koromantzos PA, Makrilakis K, Dereka X, Katsilambros N, Vrotsos IA, Madianos PN. A randomized, controlled trial on the effect of non-surgical periodontal therapy in patients with type 2 diabetes. Part I: effect on periodontal status and glycaemic control. *J Clin Periodontol*. 2011;38(2):142-147.
15. Koromantzos PA, Makrilakis K, Dereka X, et al. Effect of non-surgical periodontal therapy on C-reactive protein, oxidative stress, and matrix metalloproteinase (MMP)-9 and MMP-2 levels in patients with type 2 diabetes: a randomized controlled study. *J Periodontol*. 2012;83(1):3-10.
16. Masi S, Orlandi M, Parkar M, et al. Mitochondrial oxidative stress, endothelial function and metabolic control in patients with type II diabetes and periodontitis: A randomised controlled clinical trial. *Int J Cardiol*. 2018;271:263-268.
17. Mauri-Obradors E, Merlos A, Estrugo-Devesa A, Jané-Salas E, López-López J, Viñas M. Benefits of non-surgical periodontal treatment in patients with type 2 diabetes mellitus and chronic periodontitis: A randomized controlled trial. *J Clin Periodontol*. 2018;45(3):345-353.
18. Mizuno H, Ekuni D, Maruyama T, et al. The effects of non-surgical periodontal treatment on glycemic control, oxidative stress balance and quality of life in patients with type 2 diabetes: A randomized clinical trial. *PLoS One*. 2017;12(11):e0188171.
19. Moeintaghavi A, Arab HR, Bozorgnia Y, Kianoush K, Alizadeh M. Non-surgical periodontal therapy affects metabolic control in diabetics: a randomized controlled clinical trial. *Aust Dent J*. 2012;57(1):31-37.
20. Pham TAV, Nguyen PA, Tran TTP, Nguyen VTT. Nonsurgical periodontal treatment improved the type 2 diabetes mellitus status in smokers: A randomized controlled trial. *Diabetes Res Clin Pract*. 2022;194:110150.
21. Nishioka S, Maruyama K, Tanigawa T, et al. Effect of non-surgical periodontal therapy on insulin resistance and insulin sensitivity among individuals with borderline diabetes: A randomized controlled trial. *J Dent*. 2019;85:18-24.
22. Raman RPC, Taiyeb-Ali TB, Chan SP, Chinna K, Vaithilingam RD. Effect of nonsurgical periodontal therapy verses oral hygiene instructions on type 2 diabetes subjects with chronic periodontitis: a randomised clinical trial. *BMC Oral Health*. 2014;14:79.
23. Rapone B, Ferrara E, Corsalini M, et al. Inflammatory status and glycemic control level of patients with type 2 diabetes and periodontitis: A Randomized Clinical Trial. *Int J Environ Res Public Health*. 2021;18(6). doi:10.3390/ijerph18063018
24. Sun WL, Chen LL, Zhang SZ, Wu YM, Ren YZ, Qin GM. Inflammatory cytokines, adiponectin, insulin resistance and metabolic control after periodontal intervention in patients with type 2 diabetes and chronic periodontitis. *Intern Med*. 2011;50(15):1569-1574.
25. Syed NK. Effects of nonsurgical periodontal therapy on glycemic control in diabetic patients under systemic administration of antidiabetic ayurvedic drug. *J Contemp Dent Pract*. 2023;24(7):481-484.
26. Tran TT, Ngo QTT, Tran DH, Nguyen TDT. Effect of two nonsurgical periodontal treatment modalities in type 2 diabetes mellitus patients with chronic periodontitis: A randomized clinical trial. *J Contemp Dent Pract*. 2021;22(11):1275-1280.
27. Tsobgny-Tsague NF, Lontchi-Yimagou E, Nana ARN, et al. Effects of nonsurgical periodontal treatment on glycated haemoglobin on type 2 diabetes patients (PARODIA 1 study): a randomized controlled trial in a sub-Saharan Africa population. *BMC Oral Health*. 2018;18(1):28.
28. Vergnes JN, Canceill T, Vinel A, et al. The effects of periodontal treatment on diabetic patients: The DIAPERIO randomized controlled trial. *J Clin Periodontol*. 2018;45(10):1150-1163.
29. Wu Y, Chen L, Wei B, Luo K, Yan F. Effect of non-surgical periodontal treatment on visfatin concentrations in serum and gingival crevicular fluid of patients with chronic periodontitis and type 2 diabetes mellitus. *J Periodontol*. 2015;86(6):795-800.
30. Yun F, Firkova EI, Jun-Qi L, Xun H. Effect of non-surgical periodontal therapy on patients with type 2 diabetes mellitus. *Folia Med* . 2007;49(1-2):32-36.
31. Wang S, Liu J, Zhang J, et al. Glycemic control and adipokines after periodontal therapy in patients with Type 2 diabetes and chronic periodontitis. *Braz Oral Res*. 2017;31:e90.
32. Wang Y, Liu HN, Zhen Z, et al. A randomized controlled trial of the effects of non-surgical periodontal therapy on cardiac function assessed by echocardiography in type 2 diabetic patients. *J Clin Periodontol*. 2020;47(6):726-736.
33. Caneiro-Queija L, López-Carral J, Martin-Lancharro P, Limeres-Posse J, Diz-Dios P, Blanco-Carrion J. Non-surgical treatment of periodontal disease in a pregnant Caucasian women population: Adverse Pregnancy Outcomes of a Randomized Clinical Trial. *Int J Environ Res Public Health*. 2019;16(19). doi:10.3390/ijerph16193638
34. Fiorini T, Susin C, da Rocha JM, et al. Effect of nonsurgical periodontal therapy on serum and gingival crevicular fluid cytokine levels during pregnancy and postpartum. *J Periodontal Res*. 2013;48(1):126-133.
35. Jeffcoat MK, Hauth JC, Geurs NC, et al. Periodontal disease and preterm birth: results of a pilot intervention study. *J Periodontol*. 2003;74(8):1214-1218.
36. López NJ, Smith PC, Gutierrez J. Periodontal therapy may reduce the risk of preterm low birth weight in women with periodontal disease: a randomized controlled trial. *J Periodontol*. 2002;73(8):911-924.
37. Macones GA, Parry S, Nelson DB, et al. Treatment of localized periodontal disease in pregnancy does not reduce the occurrence of preterm birth: results from the Periodontal Infections and Prematurity Study (PIPS). *Am J Obstet Gynecol*. 2010;202(2):147.e1-8.
38. Michalowicz BS, Novak MJ, Hodges JS, et al. Serum inflammatory mediators in pregnancy: changes after periodontal treatment and association with pregnancy outcomes. *J Periodontol*. 2009;80(11):1731-1741.
39. Michalowicz BS, Hodges JS, DiAngelis AJ, et al. Treatment of periodontal disease and the risk of preterm birth. *N Engl J Med*. 2006;355(18):1885-1894.
40. Michalowicz BS, DiAngelis AJ, Novak MJ, et al. Examining the safety of dental treatment in pregnant women. *J Am Dent Assoc*. 2008;139(6):685-695.
41. Newnham JP, Newnham IA, Ball CM, et al. Treatment of periodontal disease during pregnancy: a randomized controlled trial. *Obstet Gynecol*. 2009;114(6):1239-1248.
42. Offenbacher S, Beck JD, Jared HL, et al. Effects of periodontal therapy on rate of preterm delivery: a randomized controlled trial. *Obstet Gynecol*. 2009;114(3):551-559.
43. Offenbacher S, Beck J, Jared H, et al. 3: Maternal oral therapy to reduce obstetric risk (MOTOR): A report of a multi-centered periodontal therapy randomized-controlled trial on rate of preterm delivery. *Am J Obstet Gynecol*. 2008;199(6):S2.
44. Oliveira AMSD, de Oliveira PAD, Cota LOM, Magalhães CS, Moreira AN, Costa FO. Periodontal therapy and risk for adverse pregnancy outcomes. *Clin Oral Investig*. 2011;15(5):609-615.
45. Penova-Veselinovic B, Keelan JA, Wang CA, Newnham JP, Pennell CE. Changes in inflammatory mediators in gingival crevicular fluid following periodontal disease treatment in pregnancy: relationship to adverse pregnancy outcome. *J Reprod Immunol*. 2015;112:1-10.
46. Pirie M, Linden G, Irwin C. Intrapregnancy non-surgical periodontal treatment and pregnancy outcome: a randomized controlled trial. *J Periodontol*. 2013;84(10):1391-1400.
47. Radnai M, Pál A, Novák T, Urbán E, Eller J, Gorzó I. Benefits of periodontal therapy when preterm birth threatens. *J Dent Res*. 2009;88(3):280-284.
48. Reddy BVR, Tanneeru S, Chava VK. The effect of phase-I periodontal therapy on pregnancy outcome in chronic periodontitis patients. *J Obstet Gynaecol*. 2014;34(1):29-32.
49. Sadatmansouri S, Sedighpoor N, Aghaloo M. Effects of periodontal treatment phase I on birth term and birth weight. *J Indian Soc Pedod Prev Dent*. 2006;24(1):23-26.
50. Tarannum F, Faizuddin M. Effect of periodontal therapy on pregnancy outcome in women affected by periodontitis. *J Periodontol*. 2007;78(11):2095-2103.
51. Weidlich P, Moreira CHC, Fiorini T, et al. Effect of nonsurgical periodontal therapy and strict plaque control on preterm/low birth weight: a randomized controlled clinical trial. *Clin Oral Investig*. 2013;17(1):37-44.
52. Beck JD, Couper DJ, Falkner KL, et al. The Periodontitis and Vascular Events (PAVE) pilot study: adverse events. *J Periodontol*. 2008;79(1):90-96.
53. Bokhari SAH, Khan AA, Butt AK, et al. Non-surgical periodontal therapy reduces coronary heart disease risk markers: a randomized controlled trial. *J Clin Periodontol*. 2012;39(11):1065-1074.
54. Caúla AL, Lira-Junior R, Tinoco EMB, Fischer RG. The effect of periodontal therapy on cardiovascular risk markers: a 6-month randomized clinical trial. *J Clin Periodontol*. 2014;41(9):875-882.
55. Czesnikiewicz-Guzik M, Osmenda G, Siedlinski M, et al. Causal association between periodontitis and hypertension: evidence from Mendelian randomization and a randomized controlled trial of non-surgical periodontal therapy. *Eur Heart J*. Published online September 1, 2019. doi:10.1093/eurheartj/ehz646
56. Hada DS, Garg S, Ramteke GB, Ratre MS. Effect of non-surgical periodontal treatment on clinical and biochemical risk markers of cardiovascular disease: A randomized trial. *J Periodontol*. 2015;86(11):1201-1211.
57. Ide M, McPartlin D, Coward PY, Crook M, Lumb P, Wilson RF. Effect of treatment of chronic periodontitis on levels of serum markers of acute-phase inflammatory and vascular responses. *J Clin Periodontol*. 2003;30(4):334-340.
58. Lobo MG, Schmidt MM, Lopes RD, et al. Treating periodontal disease in patients with myocardial infarction: A randomized clinical trial. *Eur J Intern Med*. 2020;71:76-80.
59. Montenegro MM, Ribeiro IWJ, Kampits C, et al. Randomized controlled trial of the effect of periodontal treatment on cardiovascular risk biomarkers in patients with stable coronary artery disease: Preliminary findings of 3 months. *J Clin Periodontol*. 2019;46(3):321-331.
60. Offenbacher S, Beck JD, Moss K, et al. Results from the Periodontitis and Vascular Events (PAVE) Study: a pilot multicentered, randomized, controlled trial to study effects of periodontal therapy in a secondary prevention model of cardiovascular disease. *J Periodontol*. 2009;80(2):190-201.
61. Rapone B, Ferrara E, Qorri E, et al. The Impact of Periodontal Inflammation on Endothelial Function Assessed by Circulating Levels of Asymmetric Dimethylarginine: A Single-Blinded Randomized Clinical Trial. *J Clin Med Res*. 2022;11(14). doi:10.3390/jcm11144173
62. Saffi MAL, Rabelo-Silva ER, Polanczyk CA, et al. Periodontal therapy and endothelial function in coronary artery disease: A randomized controlled trial. *Oral Dis*. 2018;24(7):1349-1357.
63. Seinost G, Horina A, Arefnia B, et al. Periodontal treatment and vascular inflammation in patients with advanced peripheral arterial disease: A randomized controlled trial. *Atherosclerosis*. 2020;313:60-69.
64. Sen S, Curtis J, Hicklin D, et al. Periodontal Disease Treatment After Stroke or Transient Ischemic Attack: The PREMIERS Study, a Randomized Clinical Trial. *Stroke*. 2023;54(9):2214-2222.
65. Taylor B, Tofler G, Morel-Kopp MC, et al. The effect of initial treatment of periodontitis on systemic markers of inflammation and cardiovascular risk: a randomized controlled trial. *Eur J Oral Sci*. 2010;118(4):350-356.
66. Tonetti MS, D’Aiuto F, Nibali L, et al. Treatment of periodontitis and endothelial function. *N Engl J Med*. 2007;356(9):911-920.
67. Vidal F, Figueredo CMS, Cordovil I, Fischer RG. Periodontal therapy reduces plasma levels of interleukin-6, C-reactive protein, and fibrinogen in patients with severe periodontitis and refractory arterial hypertension. *J Periodontol*. 2009;80(5):786-791.
68. Zhou QB, Xia WH, Ren J, et al. Effect of intensive periodontal therapy on blood pressure and endothelial microparticles in patients with prehypertension and periodontitis: A randomized controlled trial. *J Periodontol*. 2017;88(8):711-722.
69. Isola G, Tartaglia GM, Santonocito S, Polizzi A, Williams RC, Iorio-Siciliano V. Impact of N-terminal pro-B-type natriuretic peptide and related inflammatory biomarkers on periodontal treatment outcomes in patients with periodontitis: An explorative human randomized-controlled clinical trial. *J Periodontol*. 2023;94(12):1414-1424.
70. Fu YW, Li XX, Xu HZ, Gong YQ, Yang Y. Effects of periodontal therapy on serum lipid profile and proinflammatory cytokines in patients with hyperlipidemia: a randomized controlled trial. *Clin Oral Investig*. 2016;20(6):1263-1269.
71. Oz SG, Fentoglu O, Kilicarslan A, et al. Beneficial effects of periodontal treatment on metabolic control of hypercholesterolemia. *South Med J*. 2007;100(7):686-691.
72. Chung WC, Kao CC, Huang CF, Lee CY, Lu HK, Wu MS. Effects of Periodontal Treatment in Patients with Periodontitis and Kidney Failure: A Pilot Study. *Int J Environ Res Public Health*. 2022;19(3). doi:10.3390/ijerph19031533
73. Fang F, Wu B, Qu Q, et al. The clinical response and systemic effects of non-surgical periodontal therapy in end-stage renal disease patients: a 6-month randomized controlled clinical trial. *J Clin Periodontol*. 2015;42(6):537-546.
74. Vachhani KS, Bhavsar NV. Effects of non-surgical periodontal therapy on serum inflammatory factor high-sensitive C-reactive protein, periodontal parameters and renal biomarkers in patients with chronic periodontitis and chronic kidney disease. *Dent Med Probl*. 2021;58(4):489-498.
75. Wehmeyer MMH, Kshirsagar AV, Barros SP, et al. A randomized controlled trial of intensive periodontal therapy on metabolic and inflammatory markers in patients With ESRD: results of an exploratory study. *Am J Kidney Dis*. 2013;61(3):450-458.
76. Al-Katma MK, Bissada NF, Bordeaux JM, Sue J, Askari AD. Control of periodontal infection reduces the severity of active rheumatoid arthritis. *J Clin Rheumatol*. 2007;13(3):134-137.
77. Nguyen VB, Nguyen TT, Huynh NCN, Nguyen KD, Le TA, Hoang HT. Effects of non-surgical periodontal treatment in rheumatoid arthritis patients: A randomized clinical trial. *Dent Med Probl*. 2021;58(1):97-105.
78. Thilagar S, Theyagarajan R, Mugri MH, et al. Periodontal Treatment for Chronic Periodontitis With Rheumatoid Arthritis. *Int Dent J*. 2022;72(6):832-838.
79. Basher SS, Saub R, Vaithilingam RD, et al. Impact of non-surgical periodontal therapy on OHRQoL in an obese population, a randomised control trial. *Health Qual Life Outcomes*. 2017;15(1):225.
80. Milanesi FC, Greggianin BF, Dos Santos GO, et al. Effect of periodontal treatment on glycated haemoglobin and metabolic syndrome parameters: A randomized clinical trial. *J Clin Periodontol*. 2023;50(1):11-21.
81. Montero E, López M, Vidal H, et al. Impact of periodontal therapy on systemic markers of inflammation in patients with metabolic syndrome: A randomized clinical trial. *Diabetes Obes Metab*. 2020;22(11):2120-2132.
82. Maybodi FR, Bashiri H, Sezavar K, Owlia F. Effect of periodontal treatment on serum inflammatory parameters and disease activity in patients with systemic lupus erythematosus: A randomized controlled trial. *J Indian Soc Periodontol*. 2022;26(6):564-569.

**Supplemental Figure 1 – Study coding sheet developed and utilized for data extraction**

**Data extraction coding sheet**

*Study Info:*

PMID #: ______________________________

Name of Journal: _______________________

Study year: ____________________________

First author last name: ___________________

Main disease area of focus: (circle/highlight one)

CVD Diabetes (Type 1; Type2; Prediabetes) ABO CKD Obesity

RA OTHER (specify) _____________

Study setting:

Academic Hospital/Clinical Both Other:____________

Study country: _______________

Reported funding source: Yes No Unclear

*Sample:*

Total sample size:_____________

Did treatment and control have the same characteristics? (Circle/highlight one)

Yes No

Treatment group sample size:_________

Number loss to follow-up:___________

Control group sample size: _______________

Number loss to follow-up: ___________

Race/Ethnicities represented: White Black Asian/Pacific Islander American Indian/Alaska Native

Other Hispanic Non-Hispanic NS (non-specified)

Sex represented (Circle/highlight one): Male Female Both NS

*Periodontal disease description:*

Periodontal disease definition:

Extent of periodontal disease: (Circle/highlight one)

Generalized/Chronic Localized /Acute Both NS

Short periodontal disease definition: (Circle/highlight one)

Not explicitly defined (1)

Mild periodontitis (2)

Mild to moderate periodontitis (3)

Moderate periodontitis (4)

Moderate to severe periodontitis (5)

Severe periodontitis (6)

Mild to severe periodontitis (7)

Other (8)

*Intervention:*

Did study have true control group (participants who received no scaling and root planing)? (Circle/highlight one)

Yes No

Did study consider smoking status of study participants? (Circle/highlight one)

Yes No

*(Circle/Highlight the group which received interventions listed below)*

**Intervention: Treatment Group Control Group**

Mechanical debridement/scaling and root planning (SRP)/subgingival scaling T C

Ultrasonic debridement T C

Supragingival scaling T C

Prophylaxis/Polish T C

Oral hygiene instructions T C

Extractions T C

Restorations T C

Medications T C

Describe medications administered to treatment group:

Describe medications administered to control group:

Any other interventions described?___________________________________________________________________________

Duration of study (Time from treatment to re-evaluation):__________

If **ABO** focus, what week of gestation was treatment administered? _________

If **Diabetes** focus, what was baseline HbA1c for included patients (average/inclusion criteria range)? _________

Overall general conclusion as described in study abstract:

SRP is beneficial SRP is detrimental/adverse No difference as result of SRP

*Additional Notes:*

*Outcomes at each follow-up time-period:*

| **OUTCOME STUDIED** | **RESULT** | **EFFECT** |
| --- | --- | --- |
| EX: IL-6 | No change | Null |
| EX: CRP | Decrease | Beneficial |
|  |  |  |
|  |  |  |
|  |  |  |
|  |  |  |
|  |  |  |
|  |  |  |
|  |  |  |
|  |  |  |
|  |  |  |
|  |  |  |
|  |  |  |
|  |  |  |
|  |  |  |
|  |  |  |
|  |  |  |
|  |  |  |
|  |  |  |
|  |  |  |
|  |  |  |

- End of Coding Sheet -
